# Consequences of Early Maternal Deprivation on Neuroinflammation and Mitochondrial Dynamics in the Central Nervous System of Male and Female Rats

**DOI:** 10.3390/biology13121011

**Published:** 2024-12-04

**Authors:** Diego San Felipe, Beatriz Martín-Sánchez, Khaoula Zekri-Nechar, Marta Moya, Ricardo Llorente, Jose J. Zamorano-León, Eva M. Marco, Meritxell López-Gallardo

**Affiliations:** 1 Department of Physiology, School of Medicine, Complutense University of Madrid, Pza. Ramón y Cajal s/n, Ciudad Universitaria, 28040 Madrid, Spain; diesanfe@ucm.es (D.S.F.); beatrm14@ucm.es (B.M.-S.); martamoyamontes@ucm.es (M.M.); ricarllo@ucm.es (R.L.); mlopezga@ucm.es (M.L.-G.); 2 Instituto de Investigación Sanitaria del Hospital Clínico San Carlos (IdISSC), Department of Public Health and Maternal-Child Health, School of Medicine, Complutense University of Madrid, Pza. Ramón y Cajal s/n, Ciudad Universitaria, 28040 Madrid, Spain; kzekri@ucm.es; 3 Department of Genetics, Physiology and Microbiology, Faculty of Biological Sciences, Complutense University of Madrid, C/José Antonio Novais 12, 28040 Madrid, Spain

**Keywords:** early life stress (ELS), cytokines, citrate synthase, complex IV, hippocampal formation, inflammatory processes, mitochondria, neonatal, prefrontal cortex, sex dimorphism

## Abstract

Early life stress (ELS) is associated with an increased risk for neuropsychiatric disorders, and both neuroinflammation and mitochondrial dysfunction seem to be central to mental health. Herein, using an animal model of ELS, a single episode of maternal deprivation (MD, 24 h on pnd 9) extensively documented to elicit behavioural anomalies in male and female Wistar rats, we investigated its consequences in terms of neuroinflammation and mitochondrial dynamics in the prefrontal cortex (PFC) and the hippocampal formation (HCF). MD differentially affected the brain content of cytokines: MD induced a transient increase in pro-inflammatory cytokines (IL-1β and IL-6) in the PFC, as well as in the levels of the anti-inflammatory cytokine IL-10 in the HCF. MD also induced a significant decrease mitochondria citrate synthase activity, but MD did not exert significant changes in mitochondria Complex IV activity, revealing a generalized decrease in mitochondrial density without any change in mitochondrial respiration. In the present study, we demonstrate that MD induces neuroinflammatory processes in specific brain regions. Additional research is needed to better understand the temporal pattern of such changes, their impact on the developing brain, and their participation in the already well-known behavioural consequences of MD.

## Simple Summary

Early life stress (ELS) is a risk factor for neuropsychiatric disorders, and both neuroinflammation and mitochondrial dysfunction are linked to mental health. This study aimed to explore the short-term effects of ELS on neuroinflammation and mitochondrial dynamics using an animal model of maternal deprivation (MD) in male and female Wistar rats. MD led to a temporary increase in pro-inflammatory cytokines in the pre-frontal cortex (PFC) and anti-inflammatory cytokine levels in the hippocampal formation (HCF). It also caused a reduction in mitochondrial density, although respiratory function remained unchanged. The study found that PINK and Parkin may be involved in the MD response, with PINK expression changes observed in the hippocampus, while Parkin’s role appeared sex-dependent and potentially linked to both mitochondrial dynamics and the immune system. Further research is needed to understand the gender differences in how neuroinflammation and mitochondrial dynamics interact during brain development following MD.

## 1. Introduction

Mental disorders are a serious public health concern, causing 125 million disability-adjusted life-years globally in 2019. Gender has emerged as a critical factor for understanding mental health, since remarkable sex differences have been reported in susceptibility, chronicity, and symptomatology of psychiatric disorders. Depression and anxiety disorders are a more frequent diagnosis among women, while substance abuse and psychotic disorders are more common among men [1,2].

Among the wide variety of factors that seem to increase the risk of psychopathology, Early Life Stress (ELS) in the form of childhood maltreatment, particularly if involving physical abuse and emotional neglect, has emerged as one of the more relevant [3]. However, the influence of gender on the consequences of ELS is still an issue under discussion [4]. Therefore, the use of animal models emerges as a valuable aid to disentangle the neurobiological consequences of ELS, which may account, at least in part, for the sex-dependent vulnerability to psychopathology already reported among women and men.

Among the wide variety of animal models of ELS available, maternal deprivation (MD) was chosen in terms of neglect, as it consists of the disruption of the mother–infant interaction for 24 h, as well as given our extensive experience with the model (see [5] for review). MD has been reported to induce behavioural anomalies that resemble those observed in schizophrenic and/or depressed patients [5,6]. Researchers have not only evaluated animals’ altered phenotype but also possible neurobiological mechanisms revealing important changes in the hypothalamic–pituitary axis [7,8], on neuronal, glial, or plasticity markers and/or on the endocannabinoid system [9,10], among others. Despite most studies focusing on the outcomes of MD on adult animals [5,7,8,9,10], and only a few have studied earlier stages of life (e.g., adolescence or infancy) [11,12,13]; investigating the short-term effects of MD may provide more relevant knowledge for understanding the anomalous developmental niche that leads to the already described abnormal phenotype in adulthood.

Neuroinflammation has been reported to underlie some aspects of neuropsychiatric disorders. Inflammatory responses seem to elicit a strong influence on the initiation and development of several neuropsychiatric disorders, and alterations in the cytokine profile have been described in several neuropsychiatric disorders, sometimes associated with a cognitive dysfunction [14,15,16]. As indicated above, ELS has been reported to increase the risk of psychiatric disorders, and neuroinflammation might be at its base [17,18]. As confirmation of previous evidence, results from a meta-analysis demonstrated a pro-inflammatory status in adults with a history of childhood trauma [19]. Indeed, ELS has been reported to persistently alter immune reactivity within the brain, thus interfering with cognitive function and mental health at all stages of life [20]. Increases in some pro-inflammatory markers, such as IL-1β, IL-6, and TNFα, have been detected in several brain areas of rodents following diverse ELS protocols [21,22,23], and IL-1β has been given a central role in the priming effects of MD [24].

Interestingly, there is an increasing body of evidence suggesting a relationship between neuropsychiatric disorders and brain metabolism dysfunction [25,26,27]. Mitochondria play a critical role in the generation of cellular metabolic energy, and, moreover, mitochondrial damage has been suggested to be an important factor in the pathogenesis of a range of psychiatric disorders, including schizophrenia, depression, and bipolar disorder [28,29]. Mitochondria are not only crucial to producing energy through the mitochondrial respiratory chain but are also key in other cellular reactions such as the inflammatory response and production of reactive oxygen species (ROS), which have been proposed as a mechanistic pathway linking childhood adversity and mental health both in animal and human models [27,30]. Mitochondrial functionality is preserved mainly by a process called mitophagy, which allows the removal of damaged mitochondria [31]. Recently, adequate mitophagy has been related to neuroprotective and anti-inflammatory phenotypes [27]. However, an inadequate capacity of mitophagy to remove damaged mitochondria leads to the accumulation of damaged mitochondria and thus to impaired mitochondrial homeostasis [32]. Dysregulation of mitochondrial dynamics has been identified as one key pathogenic mechanism in a diversity of diseases and pathologies, including neurodegenerative disorders [33,34]. At present, the involvement of mitochondrial impairments in some psychiatric disorders seems to be solid [25,26,27,28,29,30]; however, research remains to be performed in the context of ELS.

Taking all together, since both brain neuroinflammation and mitophagy have been identified in several neurodegenerative and neuropsychiatric disorders, we aimed to investigate these processes in the MD animal model, as ELS has emerged as a critical risk factor for psychopathology. Notably, a gender perspective has been considered in the present study, given that sex bias is still present in clinical and preclinical research of neuropsychiatric disorders [35,36]. Therefore, by using a single episode of MD (24 h on pnd 9), we aimed to investigate sex-dependent consequences on brain neuroinflammation by (1) measuring in a multiplex analysis several pro- and anti-inflammatory cytokines and chemokines; as well as (2) mitochondrial function and expression of proteins involved in mitophagy. Our investigation focused on specific brain regions particularly vulnerable to stress and critically involved in the control of emotional homeostasis and cognition, i.e., the prefrontal cortex (PFC) and hippocampal formation (HCF).

## 2. Materials and Methods

The present study was carried out in accordance with the European Directive 2010/63/EU and in compliance with the Spanish Royal Decree 53/2013 on the protection of animals used for research and other scientific purposes. The protocol was also approved by the Local Ethics Committee of the Complutense University of Madrid (Madrid, Spain): Ref. PROEX 006/19. This work has been written following the ARRIVE guidelines (Animal Research: Reporting of In Vivo Experiments), which contain the recommendations to describe and publish the studies carried out using animals for experimental purposes.

### 2.1. Animals

Experimental subjects were the offspring of adult *Wistar* rats (*Rattus norvegicus*) purchased from Janvier Labs^®^ (Le Genest-Saint-Isle, France). Animals were always housed in polycarbonate cages (55 × 32 × 18 cm) at the animal facilities of the School of Medicine, Complutense University of Madrid (ES28079000086). Animals were maintained at constant conditions (temperature, 21 ± 1 °C and humidity, 60 ± 10%), under a 12 h light-dark inverted cycle (lights on at 20.00 h), with ad libitum access to a control pelleted diet (Altromin^®,^ Lage, Nordrhein-Westfalen, Germany) and water. Following 15 days of habituation, animals were mated (one male with two females) for five consecutive days. Then, pregnant females were isolated and daily observed to check for the delivery day, which was registered as postnatal day 0 (pnd 0). On the same day, litters were culled, and sex was balanced up to 8 pups per dam (each litter containing 4 male and 4 female pups); no cross-fostering was allowed in this study. In the present study, animals from twelve litters were employed (N = 96 animals: 48 males and 48 females).

### 2.2. ELS Animal Model: Maternal Deprivation (MD)

We employed an early maternal deprivation (MD) model, performed according to Ellenbroek and cols. [6], as previously described by our group [7,8,9,10,11,12,13]. In brief, on pnd 9, half of the litters were submitted to 24 h of Maternal deprivation, i.e., dams were removed from their home-cages at 09.00 h and pups were left undisturbed in their home-cages, allocated in the same room, next to the cage housing their corresponding mother. No food or heating was provided to pups during the deprivation protocol. On the next day, pnd 10, at 09.00 h, dams were returned to their home-cages with their corresponding litter. Animals from the control group (CTRL) were submitted to the same manipulation except for the MD episode. Four experimental groups were formed: CTRL males and CTRL females (from 6 dams), and MD males and MD females (from another 6 dams).

### 2.3. Body Weight Evaluation

The body weight (BW) of pups was recorded throughout the study. Specifically, pups’ BW was registered at birth (pnd 0), just before and after the MD episode (pnd 9 and 10), and one day before animals’ sacrifice (pnd 12 and 19) to avoid any possible interference between the manipulation and the physiological parameters evaluated after sacrifice.

### 2.4. Evaluation of Maternal Behaviour

The maternal behaviour of each dam was observed and scored for one period of 30 min after the neonatal intervention, at pnd 10 and pnd 11. The behaviour of each dam was live scored every 3 min (11 observations per day): pup retrieval, maternal contact, licking and grooming (LG), passive nursing posture (low arch/blanket nursing), active nursing posture (middle and high arch posture), and away from the nest. Non-maternal care behaviours of the dam were also recorded: exploring, eating, drinking, chasing tail, self-grooming, digging, and sleeping. The observation of other litter conditions was also recorded: split litter, buried pups, etc. The frequency observed of each behaviour each day was determined for each litter-dam. Maternal care behaviours were based on previously existing literature [37,38,39]. For the behavioural analysis, non-maternal care behaviours were analysed in combination, as well as the categories LG, passive, and active nursing, which were collapsed into a single behavioural category called ‘maternal nursing’. The total number of litters observed was 12, with 6 from each condition (CTRL and MD).

### 2.5. Righting Reflex (RR) Test

The righting reflex (RR) test was performed on pnd 9, 10, and 12 in order to evaluate the degree of maturity and motor development of the animals [40,41]. Briefly, pups were placed flat on their back, in a supine position, and their latency time to restore a normal prone position, onto the stomach with all limbs outstretched from the body, was measured. This was considered fully achieved when the pups turned 180° around their longitudinal axis, and their four paws were in contact with the plane surface within a 30 s observation time frame [41].

### 2.6. Tissue Collection

**Figure 1:**
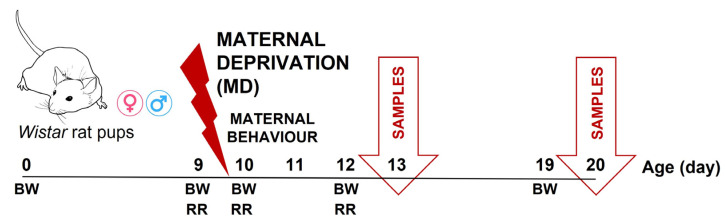
Experimental design. The male and female offspring of Wistar rats were exposed to early life stress, maternal deprivation (MD, 24 h on pnd 9). During the study, animals’ body weight (BW) was recorded, animals’ performance in the righting reflex (RR) was evaluated, maternal behaviour was observed, and animals were then sacrificed at postnatal days 13 and 20 to obtain brain samples: prefrontal cortex (PFC) and hippocampal formation (HCF).

At postnatal days 13 and 20, rats were sacrificed by anaesthetic overdose (sodium pentobarbital, 650 mg/kg, i.p., Dolethal^®^, Vetoquinol, Madrid, Spain) and decapitated; brains were rapidly extracted and dissected on ice: the prefrontal cortex (PFC) and hippocampal formation (HCF) were selected for the present study. Brain samples were weighed, flash-frozen in liquid nitrogen, and stored at −30 °C until the analysis. In the present study, only four animals per litter were used (two males and two females per litter), thus a total of 48 animals; the other half of each litter was dedicated to a complementary immunohistochemical study (data not published). A schematic experimental design is shown in Figure 1.

### 2.7. Multiplex Analysis: Cytokines and Chemokines Measurements

Inflammatory markers were evaluated in the PFC and HCF, using a Multiplex Bead Array Assay (RECYTMAG-65K-06, MILLIPLEX^®^, Merck-Millipore, Phillipsburg, NJ, USA). Multiplex Bead Array Assays are a high-performance technology that enables the quick and highly efficient quantitative evaluation of many analytes in a very small volume of tissue homogenate [42]. In this assay, we measured levels of IL-1β, IL-6, TNFα, IL-10, fractalkine (CX3CL1), and monocyte chemotactic protein monocyte chemoattractant protein-1 (CCL2/MCP-1). The manufacturer’s instructions were followed for processing and measuring the tissue. In brief, samples were diluted (1:3) in a multiplexing lysis buffer (Ref. 43-040, Merck, Phillipsburg, NJ, USA) at 4 °C, homogenized, and stored at −30 °C overnight (O/N). The next day, samples were re-suspended and centrifuged twice (4 °C, 12,000× *g*, 5 min). The assay was performed in a 96-well microplate following the manufacturer’s instructions and applying an analysis protocol described previously. This multiplex assay was performed blind to the different experimental groups in collaboration with Raffer^®^ laboratories (Zaragoza, Spain). Samples were run in duplicate. Initially, the plate was washed by adding a 200 µL aliquot of assay buffer to each well. Afterwards, 25 µL of assay buffer, 25 µL of lysis buffer, and then 25 µL of the standard or brain tissue extracts (15 mg/mL protein measured using the Bradford method, Ref. 500-0006, Bio-Rad, Hercules, CA, USA) were added to each well, as indicated. The plate was then incubated for 2 h at room temperature, the plate content was then discharged, and the wells were washed. After this protocol, 25 µL of the working bead antibodies mixture was added to each well. Incubation was again conducted in darkness, at room temperature under agitation, for 2 h. After washing, 25 μL of detection antibodies were added to each well, and the plate was incubated in darkness, at room temperature under agitation, for 1 h this time. The microspheres were then washed, and 25 µL of the streptoavidin conjugated with phycoerythrin (streptoavidin-PE) was added to each well. The plate was then incubated, as previously described, for 30 min. After washing away the non-antibody bound Streptoavidin–PE complex, 125 µL of sheath fluid was added, and microspheres were re-suspended on a plate shaker for 5 min. Quantitative data were obtained using the Luminex-100 system (Luminex Corporation, Austin, TX, USA), and data analysis was performed in Luminex xPONENT software (version 3.1) using a five-parameter logistic (5PL) regression non-linear model to obtain the concentration of each of the analytes (pg/mL). The Limit of Quantification (LOQ) of the assay was (in pg/mL) 9.77 for IL-1β, 292.97 for IL-6, 2.44 for TNFα, 29.30 for IL-10, 2.44 for fractalkine/CX3CL1, and 117.19 for MCP-1/CCL2.

### 2.8. Mitochondrial Density and Functionality: Citrate Synthase (CS) and Complex IV (CIV) Activity

Citrate synthase (CS) activity, used as a quantitative enzyme marker for the presence of mitochondria [43], was evaluated in the PFC and HCF using the Citrate Synthase Activity Colorimetric Assay Kit (K318-100, Biovision- Avantor^TM^, Exton, PA, USA). In brief, a spectrophotometric assay based on the activity of CS that catalyses the conversion of acetyl-CoA and oxaloacetate into citrate was employed. Tissue samples (10 mg) were homogenized with the kit buffer and incubated in ice for 10 min; they were then centrifuged at 4 °C, 10,260 rpm, for 5 min. Supernatants (25 µL) and an equal volume of the Assay Buffer were added to each well of the plate, followed by 50 µL of the Reaction mix. Absorbance was then measured at 412 nm (MultiskanTM FC Microplate Photometer, Thermo Fisher Scientific, Waltham, MA, USA) in kinetic mode at 25 °C for 40 min (data were registered every 5 min). All samples were run in duplicate. CS activity was calculated as a function of time by choosing two time points in the linear range. CS activity was further normalized to protein content (measured using the Bradford reagent system, 500-0006, Bio-Rad), and data are expressed as nmol/min per mg of protein.

Complex IV or cytochrome C oxidase (CIV or COX) activity, proposed as an index of mitochondrial functionality, was evaluated in the PFC and HCF, using the Complex IV Rodent Enzyme Activity Microplate Assay Kit (Ab109911, Abcam, Cambridge, UK). The kit was used following the manufacturer’s instructions. Briefly, brain samples (20 mg) were homogenized with the kit buffer; a volume of the tissue homogenate containing 5.5 mg protein/mL, calculated by using the MicroBradford reagent system (Ref. 500-0006, Bio-Rad), was mixed with the kit detergent and incubated for 30 min in ice. then, the samples were centrifuged at 4 °C, 16,000 rpm, for 20 min. Supernatant was recovered, and the tissue extract was adjusted to the recommended dilution (0.25 μg/μL). The plate was loaded and incubated at room temperature for 3 h. The plate was washed according to the protocol, and assay solution was then added to the wells. Absorbance was measured at an optical density (OD) of 550 nm in kinetic mode at 30 °C for 120 min, with data being registered every 5 min (EMax^®^Plus Microplate Reader, Molecular Devices, LLC, San Jose, CA, USA). All samples were run in duplicate. The CIV activity rate was determined by calculating the slope between two points within the linear region, and data are expressed as mOD/min per mg of protein. CIV activity was further analysed with reference to CS activity (ratio CIV/CS activity).

### 2.9. Mitophagy. Expression of Proteins Involved in Mitophagy: PINK and Parkin

Tissue samples were homogenized at 4 °C at a ratio of 1:3 (w/v) in an ice-cold lysis buffer (Ref. 43-040, Merck) and stored at −30 °C overnight. The homogenate obtained was centrifuged two times at 14,000 rpm for 5 min each, at 4 °C, and the supernatant was then collected. The total protein concentration in the supernatant per sample was calculated using the MicroBradford reagent system (Ref. 500-0006, Bio-Rad), quantified using a Multiskan FC microplate photometer (Thermo Fisher Technologies, Waltham, MA, USA) at a wavelength of 595 nm, and using the Skan-lt software for Multiskan FC, version 2.5. Brain samples were then aliquoted and stored at −30 °C until use. The amount of protein loaded was 40 μg of protein per gel line. Samples were then diluted (1:1) with a Laemmli sample buffer and heated at 100 °C for 6 min in a thermoblock (FB15103 TA 120, FALC Instruments, Fisher Scientific, Waltham, MA, USA). They were then allowed to cool at room temperature before loading onto 10% SDS-polyacrylamide gels for electrophoretic separation (15 min at 90 V, followed by 1 h and 15 min at 120 V), and were subsequently transferred (46 min at 2.5 A and 11 V at room temperature) onto nitrocellulose membranes (Amersham Potran Premium 0.45 μm NC), by a semi-dry trans-ferred system (Pierce G2 Fast Blotter, 22 Thermo Scientific). Samples from all experimental groups were run concurrently, and a colorimetric molecular mass marker, including standards ranging from 10 kDa to 245 kDa (Ref. A8889, PanReac Panreac Química S.L.U., Barcelona, Spain), was loaded onto the last lane of the gel to determine protein size.

Immunostaining of the membrane was performed by saturating non-specific sites with TTBS [TBS (NaCl; Tris, pH 7.5 1 M) with 0.1% Tween-20] with 5% BSA (bovine serum albumin, Ref. A3803-50G, Sigma-Aldrich/Merck, St. Louis, MO, USA) for 2 h at room temperature under constant stirring; after three washes of 10 min each time, the membranes were incubated with the primary antibody (see Table 1) overnight at 4 °C under constant stirring. The membranes were then washed with TTBS (three times, 10 min each) and incubated for 2 h at room temperature under stirring with the secondary antibody (see Table 1). After three 10 min washes in TTBS, immunoreactive protein bands were visualized using the enhanced chemiluminescence (ECL) detection kit (Ref. SC-2048, Santa Cruz Biotechnology, Inc., Dallas, TX, USA) according to the manufacturer’s instructions. Chemiluminescent images were visualized and captured by a digital system (iBright FL1000 Western Blot Imaging Systems, Invitrogen/Thermo Fisher Scientific, Waltham, MA, USA). The membranes were also probed using the same method with anti-β-actin (see Table 1), which was used as a loading control for normalization of the blots. The relative optical density of the blots was determined with ImageJ software (U.S. National Institutes of Health, Bethesda, MD, USA, version 1.54k) by two researchers in a blinded manner. Western blot values were corrected using the internal membrane protein control, actin. The relative optical density of the blots from the male control pnd 13 group was used as a reference (100%), and data for the other experimental groups were expressed as a percentage of control males.

### 2.10. Statistical Analysis

In general, data were analysed by using a three-way analysis of variance (ANOVA), considering sex (male or female), neonatal condition (CTRL or MD), and age (pnd 13 or pnd 20), when applicable, as independent factors. In the case of body weight gain, a repeated measures two-way ANOVA was performed. Normality and homogeneity of variance were assessed using the Shapiro–Wilk and Levene’s tests, respectively. Tukey post-hoc comparisons were performed only for significant interactions, as well as additional independent ANOVA analyses with data split by age. When necessary, nonparametric analyses were performed: Kruskal–Wallis test followed by Mann–Whitney paired comparisons. The significance level was set at *p* < 0.05 for all comparisons. Statistical analyses were performed using IBM SPSS Statistics software package, version 25.0 (IBM Corporation, New York, NY, USA). Data, in tables and figures, are presented as the mean ± standard deviation (SD). All graphs were prepared using GraphPad Prism 8 (GraphPad, Boston, MA, USA).

## 3. Results

### 3.1. Body Weight Gain

**Figure 2:**
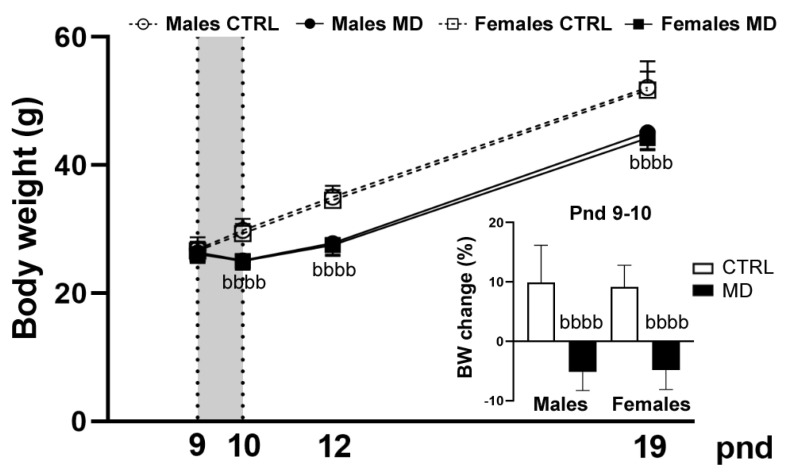
Effects of Maternal Deprivation (MD) on body weight (BW) gain from postnatal day (pnd) 9 until the end of the study. Rats were exposed to early life stress, MD (24 h on pnd 9, solid lines and symbols) or subjected to the same manipulation except for the separation period (CTRL, dashed line, and empty symbols). Pups were individually weighted before the procedure (pnd 9), immediately after (pnd 10), and on the day prior to their sacrifice (pnd 12 and 19); mean values are grouped by litter (12 individuals from three litters per experimental group). Data are presented as the mean body weight value (g) ± standard deviation (SD) of both male and female animals (Males: circles; Females: squares). As an insert: the percentage of BW changes during the deprivation period (mean ± SD). Repeated measures two−way ANOVA, followed by Tukey post-hoc comparisons: bbbb, *p* < 0.001, significant overall effect of neonatal condition.

The analysis of litters’ body weight rendered no significant effects before the neonatal stress protocol, but statistically significant differences emerged thereafter (Figure 2). In the repeated measures ANOVA, sphericity could not be assumed [Χ(9) = 32.46, *p* < 0.05]; therefore, degrees of freedom were corrected using Greenhouse–Geisser estimates of sphericity (ε = 0.46). A highly significant effect of age was found in the within-litter analysis [F(1.84, 14.75) = 1132.01, *p* < 0.001; η^2^ = 0.99], as well as a significant age by neonatal condition interaction [F(1.84, 14.75) = 17.16, *p* < 0.001; η^2^ = 0.68]. The significant effect of the neonatal condition was confirmed in the between-litter analysis [F(1,8) = 20.99, *p* = 0.002; η^2^ = 0.72], and the significant effect, absent at birth (pnd 0,) and before any manipulation (pnd 9) emerged immediately after the MD episode, pnd 10 [F(1,20) = 52.82, *p* < 0.001; η^2^ = 0.73], and persisted until the end of the present study [pnd 12: F(1,20) = 103.57, *p* < 0.001; η^2^ = 0.84; and pnd 19: F(1,8) = 17.71, *p* = 0.003; η^2^ = 0.69]. No sex differences were evidenced in body weight at this period of neonatal life.

### 3.2. Behaviour in the RR Test

Regarding the RR test conducted on pups, no differences between groups were observed in the latency time at pnd 9 [H(3) = 1.41, ns] (Table 2). However, at pnd 10, a significant difference between groups was detected [H(3) = 8.58, *p* = 0.035], indicating a statistically significant difference between CTRL and MD males (*p* = 0.04) in the absence of any further remarkable differences between groups (Table 2). Actually, at pnd 10, the percentage of pups that achieved the RR in less than 1.5 s was over 50% only among CTRL animals (CTRL males 75% and CTRL females 62.5%) compared to MD animals (only 41.7% of MD males and 37.5% of MD females). No differences in latency time were found at pnd 12 [Sex: F(1,91) = 1.86, ns; neonatal condition: F(1,91) = 0.51, ns; sex by neonatal condition: F(1,91) = 0.63, ns] (Table 2). Taken together, the MD episode induced a delay in the development of the righting reflex, determined by an increase in the time needed to acquire the normal prone position at pnd 10, immediately after the MD episode, an effect that was only evident among male animals. No sex differences in the acquisition of the RR were observed at the specific time points evaluated, even though an improvement in animals’ response over time during the evaluations was found, suggesting a developmental (growth) effect.

### 3.3. Maternal Behaviour

Maternal behaviour evaluation demonstrated a decrease in maternal contact between postnatal days 10 and 11; only a general effect of age was observed when analysing this parameter [age: F(1,20) = 15.65, *p* = 0.001; η^2^ = 0.44], in the absence of any additional effect [neonatal condition: F(1,20) = 3.18, ns; interaction between factors: F(1,20) = 0.15, ns]. Maternal nursing was independently affected by age [F(1,20) = 35.16, *p* < 0.001; η^2^ = 0.64] and by the neonatal condition [F(1,20) = 16.19, *p* = 0.001; η^2^ = 0.45], in the absence of any significant interaction between factors [F(1,20) = 0.18, ns]. As expected, maternal nursing diminished with age, whereas exposure to the MD episode significantly increased the frequency of nursing behaviours. Regarding the non-maternal care behaviours, only age affected such behaviours [F(1,20) = 5.87, *p* = 0.025; η^2^ = 0.23]; no significant effect of the neonatal condition [F(1,20) = 1.92, ns] nor significant interaction between factors [F(1,20) = 0.48, ns] was observed. The frequency of these non-maternal care behaviours increased with age (higher at pnd 11), probably to counterbalance the decrease in nursing behaviours observed during the same period (Table 3).

### 3.4. Neuroinflammatory Parameters: Cytokines and Chemokines Assay

**Figure 3:**
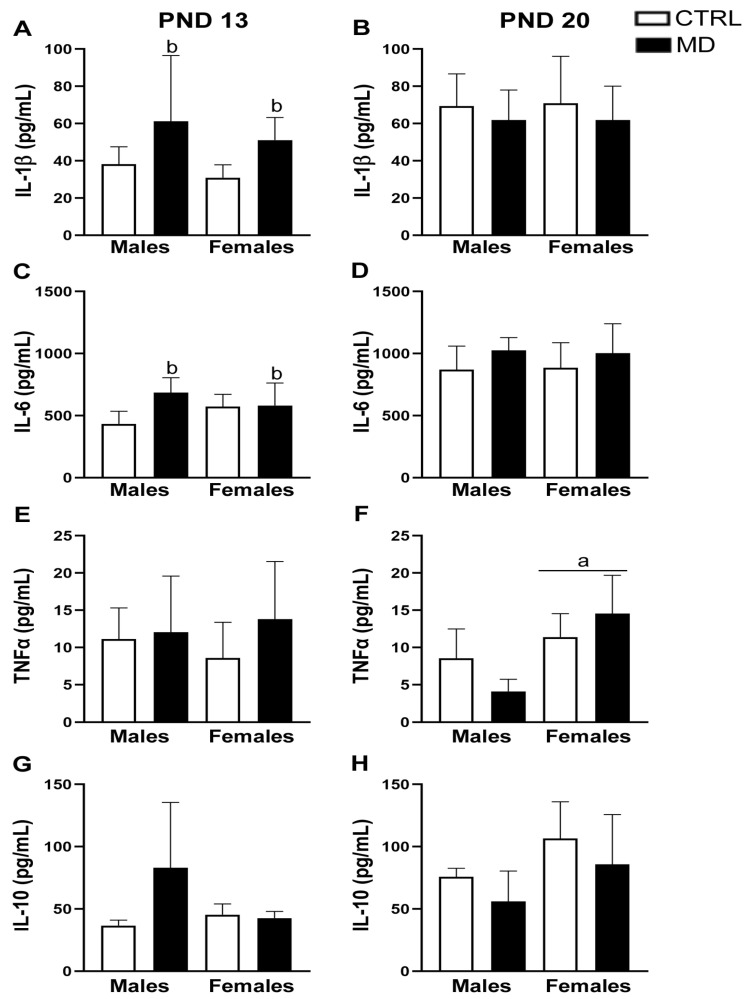
Effects of maternal deprivation (MD) on the cytokine profile of the prefrontal cortex (PFC) of rats at pnd 13 (**A**,**C**,**E**,**G**) and at pnd 20 (**B**,**D**,**F**,**H**). Histograms represent the mean ± SD (five animals per experimental group). ANOVA and Tukey post-hoc comparisons or nonparametric analysis using the Kruskal–Wallis test followed by the Mann–Whitney test: a, *p* < 0.05 significant overall effect of sex; b, *p* < 0.05 significant overall effect of MD. Age differences are explained in the text.

The main results from the multiplex analysis, although explained in detail below, are summarized in Table 4. The analysis of cortical levels of IL-1β rendered a significant general effect of age [F(1,32) = 14.89, *p* = 0.001, η^2^ = 0.32], together with a significant interaction of neonatal condition by age [F(1,32) = 6.04, *p* = 0.020, η^2^ = 0.16]. Levels of IL-1β within the PFC increased with age, from pnd 13 to pnd 20. The posterior analysis of data split by age showed that the neonatal condition induced a significant general effect only at pnd 13 [F(1,16) = 7.10, *p* = 0.017, η^2^ = 0.31], significantly increasing IL-1β levels in the PFC of both male and female animals. Such differences in IL-1β levels were transient as they disappeared by pnd 20 [neonatal condition: F(1,16) = 0.52, ns] (Figure 3A,B). IL-6 levels in the PFC showed a significant general effect of age [F(1,30) = 51.01, *p* < 0.001, η^2^ = 0.63] as well as a significant general effect of the neonatal condition [F(1,30) = 6.28, *p* = 0.018, η^2^ = 0.17]. IL-6 levels increased with age (pnd 13 < pnd 20), and the neonatal condition increased these cytokine levels. However, a further analysis of data split by age revealed that the neonatal condition effects were only significant at pnd 13 [F(1,15) = 4.63, *p* = 0.048, η^2^ = 0.24], with effects being more evident among the male population [neonatal condition by age interaction: F(1,15) = 4.03, *p* = 0.06, η^2^ = 0.21] (Figure 3C,D). The analysis of TNFα levels within the PFC revealed a significant interaction between sex and age [F(1,29) = 4.43, *p* = 0.04, η^2^ = 0.13]; only at pnd 20 was a significant sexual dimorphism appreciated [sex: F(1,15) = 16.23, *p* = 0.001, η^2^ = 0.52], and a sex by neonatal condition interaction also appeared [F(1,15) = 5.29, *p* = 0.039, η^2^ = 0.26]. However, post-hoc comparisons revealed no significant differences between experimental groups (Figure 3E,F). Cortical levels of IL-10 significantly differed between groups [H(7) = 14.82, *p* = 0.038], but post-hoc comparisons only revealed a significant general effect of age (*p* = 0.018), with IL-10 cortical levels being higher at pnd 20. No further differences could be reported (Figure 3G,H).

Levels of fractalkine/CX3CL1 in the PFC were not modified by any of the factors investigated [pnd 13: Male CTRL: 2621.23 ± 287.59 pg/mL, Male MD: 2823.38 ± 289.34 pg/mL, Female CTRL: 2937.71 ± 139.29 pg/mL, Female MD: 2755.36 ± 331.14 pg/mL; pnd 20: Male CTRL: 2610.25 ± 446.76 pg/mL, Male MD: 2996.50 ± 214.25 pg/mL, Female CTRL: 2892.33 ± 376.72 pg/mL, Female MD: 2963.56 ± 308.96 pg/mL]. Measurement of MCP-1/CCL2 levels within the PFC only achieved detectable levels exclusively for female animals at pnd 20. This analysis, although preliminary due to the reduced amount of data obtained (n = 3, per group), revealed a significant effect of the neonatal condition [t(4) = 3.51, *p* = 0.025]: MD decreased MCP-1/CCL2 levels in females aged 20 days [at pnd 20, Female CTRL: 295.24 ± 64.11 versus Female CTRL: 150.31 ± 31.77].

**Figure 4:**
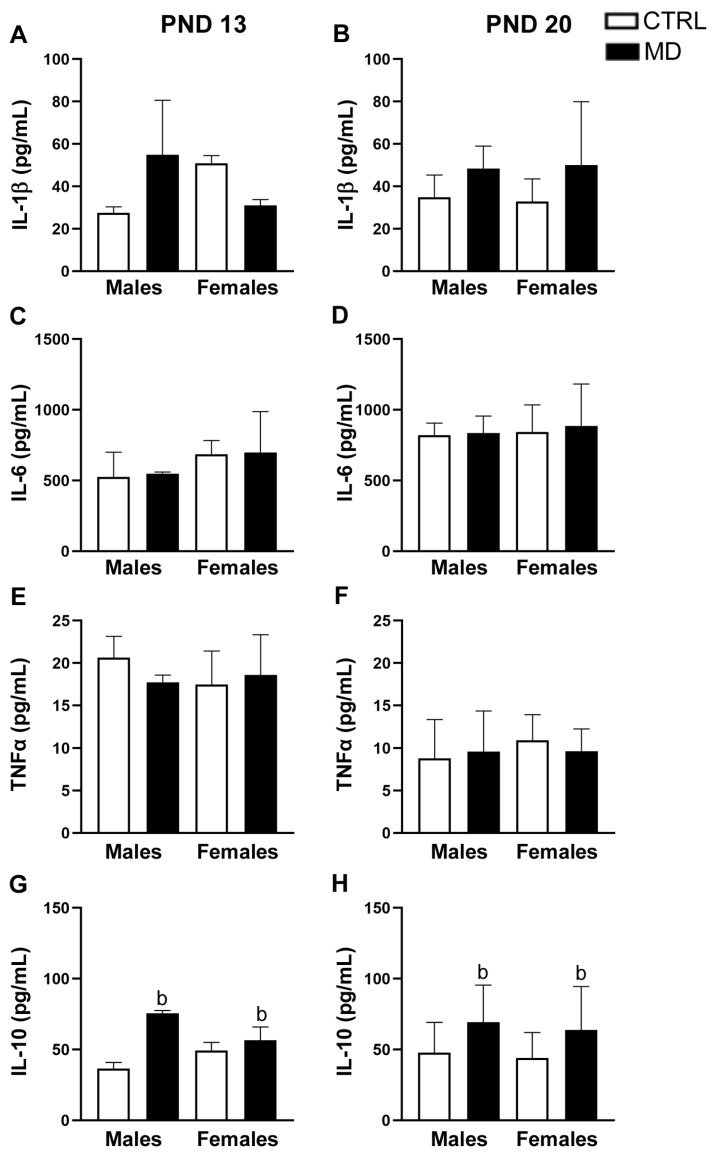
Effects of maternal deprivation (MD) on the cytokine profile of the hippocampal formation (HCF) of rats at pnd 13 (**A**,**C**,**E**,**G**) and at pnd 20 (**B**,**D**,**F**,**H**). Histograms represent the mean ± SD (5 animals per experimental group). ANOVA and Tukey post-hoc comparisons or non-parametric analysis using the Kruskal–Wallis test followed by the Mann–Whitney test: b, *p* < 0.05 significant overall effect of MD. Age differences are explained in the text.

In the HCF (Figure 4), we found no changes in the levels of IL-1β [H(7) = 10.84, ns], neither in the short term (pnd 13) nor in the long term (pnd 20) (Figure 4A,B). Hippocampal IL-6 levels were only affected by age [F(1,23) = 11.78, *p* = 0.002, η^2^ = 0.34]; levels of IL-6 within the HCF showed a general increase from pnd 13 to pnd 20 (Figure 4C,D). Similarly, TNFα levels within the HCF showed a significant general effect of age [F(1,23) = 44.59, *p* < 0.001, η^2^ = 0.66]; this cytokine in this brain region significantly decreased with neonatal development, from pnd 13 to pnd 20 (Figure 4E,F). In the HCF, the analysis of IL-10 levels revealed a significant general effect of the neonatal condition. Despite no differences between groups being achieved [H(7) = 12.11, *p* = 0.097], a posterior analysis revealed a general effect of the neonatal condition (*p* = 0.004), with IL-10 hippocampal levels showing increased levels in the MD groups; the MD increase in IL-10 levels seemed clearer at pnd 13, although a similar pattern was observed at pnd 20 despite an increased dispersion in concentration values (Figure 4G,H). Fractalkine/CX3CL1 levels were not affected by the factors analysed within the HCF [pnd 13: Male CTRL: 2263.06 ± 553.49 pg/mL, Male MD: 2288.17 ± 718.76 pg/mL, Female CTRL: 2725.14 ± 287.47 pg/mL, Female MD: 2615.19 ± 651.89 pg/mL; pnd 20: Male CTRL: 2843.76 ± 202.21 pg/mL, Male MD: 2933.99 ± 347.92 pg/mL, Female CTRL: 2699.81 ± 762.36 pg/mL, Female MD: 2574.62 ± 191.59 pg/mL]. In this brain region, results from MCP-1/CCL2 measurements could not be analysed due to the low number of samples with detectable levels.

### 3.5. Mitochondrial Function and Turnover

The main consequences of MD on specific markers of mitochondrial function and mitophagy analysed here are presented in Table 5.

#### 3.5.1. Mitochondrial Density and Function: Citrate Synthase (CS) and Complex IV (CIV) Activity

**Figure 5:**
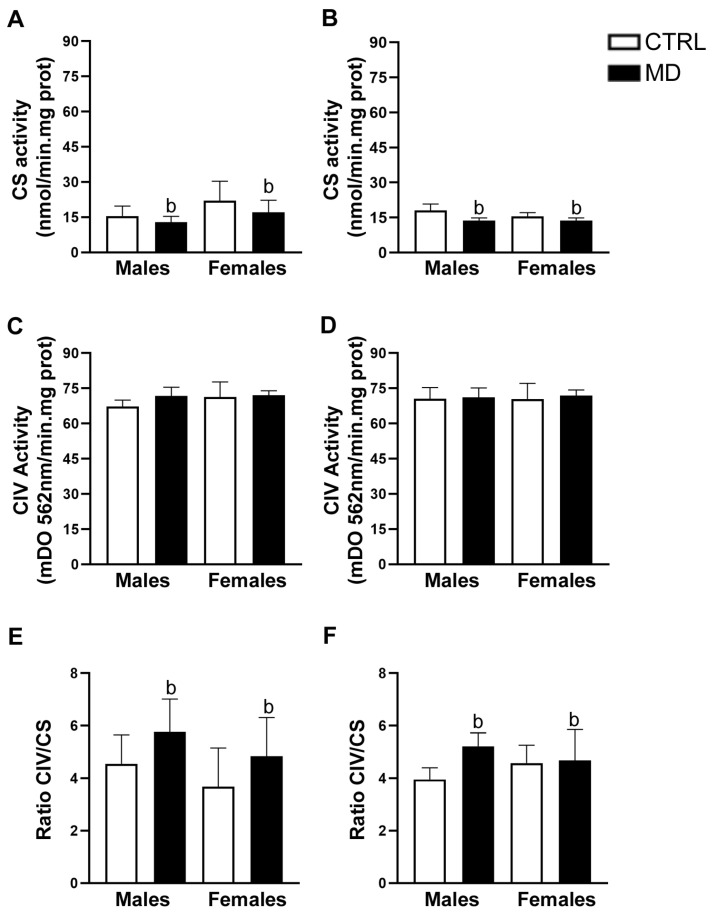
Effects of maternal deprivation (MD) on the activity of mitochondrial components of the respiratory chain, citrate synthase (CS), and complex IV (CIV) in the prefrontal cortex (PFC): CS activity at pnd 13 and 20 (**A**,**B**, respectively), CIV activity (**C**,**D**, respectively), and the activity ratio of CS relative to CIV (**E**,**F**, respectively). Histograms represent the mean ± SD (five animals per experimental group). ANOVA and Tukey post-hoc comparisons or nonparametric analysis using the Kruskal–Wallis test followed by the Mann–Whitney test: b, *p* < 0.05 significant overall effect of MD.

The results from CS activity in PFC (Figure 5) showed only a significant effect when all CTRL data were compared to all MD animals (Mann–Whitney: U = 122, *p* = 0.035). CS activity seemed to be slightly decreased by MD (Figure 5A,B). No significant effects were observed in the analysis of CIV activity within the PFC [sex: F(1,32) = 0.90, ns; neonatal condition: F(1,32) = 1.77, ns; age: F(1,32) = 0.10, ns; sex by neonatal condition: F(1,32) = 0.32, ns; sex by age: F(1,32) = 0.44, neonatal condition by age: F(1,32) = 0.33, ns; sex by neonatal condition by age: F(1,32) = 0.75, ns] (Figure 5C,D). To summarize, in the PFC, MD only induced a modest but significant decrease in CS activity, an index of mitochondrial density, but no effects in CIV activity that is related to mitochondrial activity. In addition, the ANOVA of the ratio CIV/CS revealed a statistically significant general effect of the neonatal condition [F(1,32) = 5.00, *p* = 0.032, η^2^ = 0.14] in the absence of any additional effect. Thus, the ratio CIV/CS activity was increased as a consequence of the episode of MD, regardless of sex and age (Figure 5E,F).

**Figure 6:**
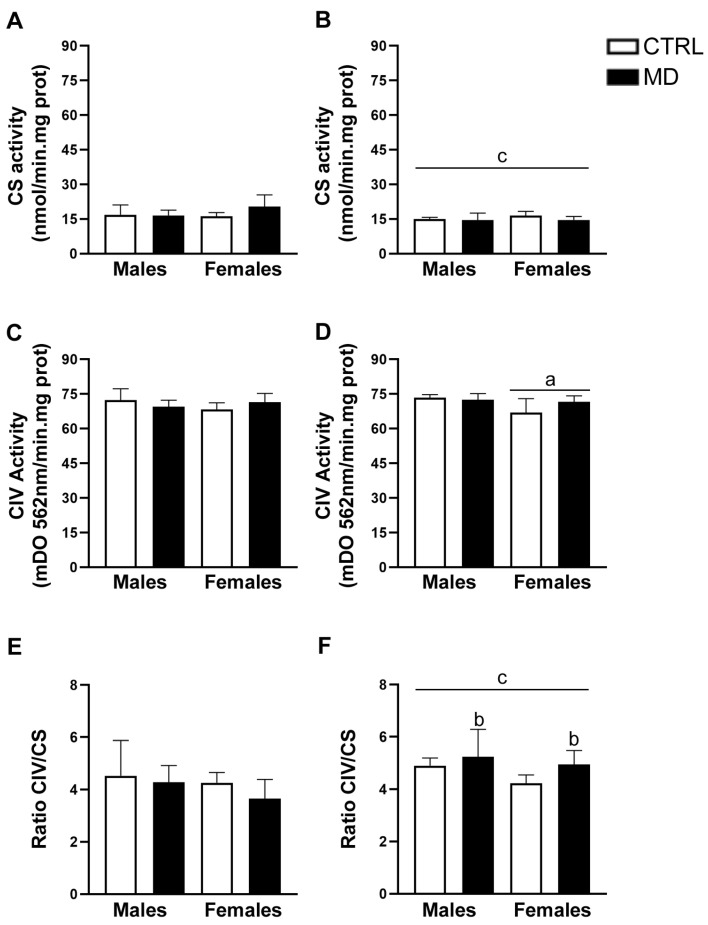
Effects of maternal deprivation (MD) on the activity of mitochondrial components of the respiratory chain, citrate synthase (CS), and complex IV (CIV) in the hippocampal formation (HF): CS activity at pnd 13 and 20 (**A**,**B**, respectively), CIV activity (**C**,**D**, respectively), and the activity ratio of CS relative to CIV (**E**,**F**, respectively). Histograms represent the mean ± SD (five animals per experimental group). ANOVA and Tukey post-hoc comparisons or nonparametric analysis using the Kruskal–Wallis test followed by the Mann–Whitney: a, *p* < 0.05 significant overall effect of sex; b, *p* < 0.05 significant overall effect of MD; c, *p* < 0.05 significant overall effect of age.

The results of CS activity within the HCF showed no significant effects (Figure 6A,B). However, further analysis of collapsed data revealed a significant effect of age [Mann–Whitney: U = 100, *p* = 0.007], with CS activity within the HCF being lower at pnd 20 compared to the earlier age (pnd 13). The analysis of CIV activity within the HCF showed a statistically significant interaction between sex and the neonatal condition [F(1,32) = 6.91, *p* = 0.013, η^2^ = 0.18] in the absence of any additional significant effect. Further analysis of data collapsed by age revealed a significant effect of sex only at pnd 20 [F(1,16) = 6.41, *p* = 0.022, η^2^ = 0.29]. Therefore, although CIV activity seemed to be lower among female animals, in general, such a sex difference only became statistically significant at pnd 20 (Figure 6C,D). The additional analysis of the ratio of CIV/CS activity within the HCF revealed a statistically significant general effect of age [F(1,32) = 8.28, *p* = 0.007, η^2^ = 0.21], with the ratio increasing with age, from pnd 13 to pnd 20. A significant interaction between the neonatal condition and age also emerged in this analysis [F(1,32) = 5.09, *p* = 0.031, η^2^ = 0.14]. Further analysis of the ratio of CIV/CS activity split by age indicated that the neonatal condition may only affect animals at pnd 20 [F(1,16) = 4.44, *p* = 0.051, η^2^ = 0.22] and not at pnd 13 [F(1,16) = 1.55, ns]. In the HCF, MD may also induce a slight increase in the ratio of CIV/CS activity, both in male and female animals, exclusively at pnd 20 (Figure 6E,F).

#### 3.5.2. Mitophagy-Related Protein Expression: PINK and Parkin

**Figure 7:**
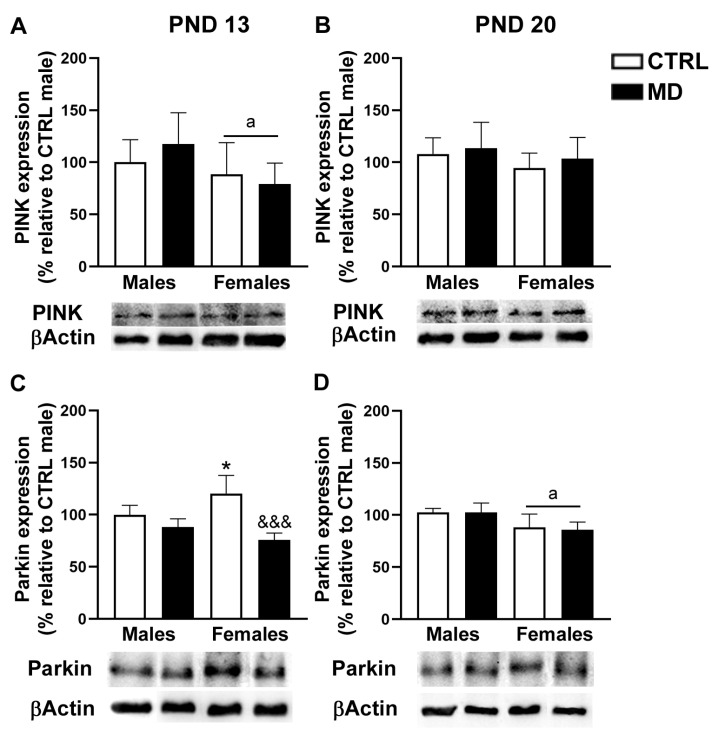
Effects of maternal deprivation (MD) on the expression levels of PINK and Parkin in the prefrontal cortex (PFC): PINK expression at pnd 13 and 20 (**A**,**B**, respectively) and Parkin expression (**C**,**D**, respectively) expressed as the change in expression relative to CTRL males. Representative images from the Western blots performed are presented under each histogram. Histograms represent the mean ± SD (four to five animals per experimental group). ANOVA and Tukey post-hoc comparisons or nonparametric analysis using the Kruskal–Wallis test followed by the Mann–Whitney: a, *p* < 0.05 significant overall effect of sex; * *p* < 0.05 vs. CTRL male; &&&, *p* < 0.005 vs. CTRL females. See “Supplementary Materials” for an example of the original Western blot images.

The analysis of PINK expression in the PFC (Figure 7A,B) revealed no more than a general sex effect [F(1,32) = 6.54, *p* = 0.016, η^2^ = 0.17] that, in a further analysis split by age, was only evident at pnd 13 [F(1,16) = 4.66, *p* = 0.046, η^2^ = 0.23], with females exhibiting a lower PINK expression than males in this brain region. The analysis of the expression of Parkin levels within the PFC (Figure 7C,D) revealed a significant interaction between factors [sex by neonatal condition by age: F(1,32) = 5.77, *p* = 0.02, η^2^ = 0.15; sex by neonatal condition: F(1,32) = 7.74, *p* = 0.009, η^2^ = 0.20; neonatal condition by age: F(1,32) = 18.24, *p* < 0.001, η^2^ = 0.36; sex by age: F(1,32) = 9.76, *p* = 0.004, η^2^ = 0.23] as well as a significant general effect of the neonatal condition [F(1,32) = 21.53, *p* < 0.001, η^2^ = 0.40]. The analysis of the Parkin expression data by age revealed, at pnd 13, a significant sex by neonatal condition interaction [F(1,16) = 11.03, *p* = 0.004, η^2^ = 0.41], together with a significant general effect of the neonatal condition [F(1,16) = 32.60, *p* < 0.001, η^2^ = 0.67]; whereas at pnd 20, only a general significant effect of the sex factor was observed [F(1,16) = 15.73, *p* = 0.001, η^2^ = 0.50]. Thus, in the short term (pnd 13), the neonatal condition decreased Parkin expression exclusively in female animals, and no effects of the neonatal condition were observed at a later age (pnd 20). Sex differences were observed in the cortical levels of Parkin expression: Females showed higher Parkin levels than males at pnd 13, but the opposite was observed at pnd 20.

**Figure 8:**
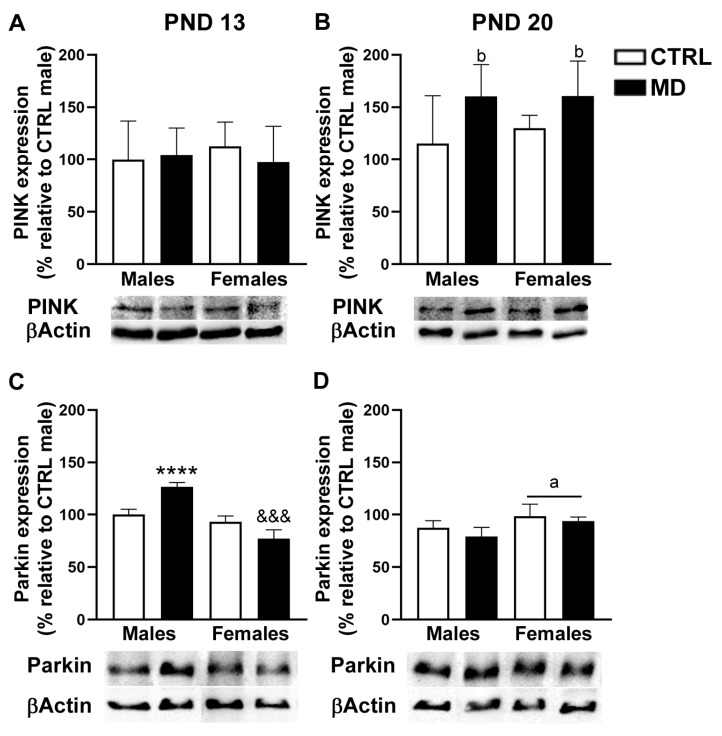
Effects of maternal deprivation (MD) on the expression levels of PINK and Parkin within the hippocampal formation (HCF): PINK expression at pnd 13 and 20 (**A**,**B**, respectively) and Parkin expression (**C**,**D**, respectively) expressed as the change in expression relative to CTRL males. Representative images from the Western blots performed are presented under each histogram. Histograms represent the mean ± SD (four to five animals per experimental group). ANOVA and Tukey post-hoc comparisons or nonparametric analysis using the Kruskal–Wallis test followed by the Mann–Whitney test: a, *p* < 0.05 significant overall effect of sex; b, *p* < 0.05 significant overall effect of MD; **** *p* < 0.001 vs. CTRL male; &&&, *p* < 0.005 vs. CTRL females. See “Supplementary Materials” for an example of the original Western blot images.

The analysis of hippocampal PINK expression (Figure 8A,B) revealed a significant interaction between neonatal condition and age [F(1,32) = 14.36, *p* = 0.001, η^2^ = 0.31], together with a significant effect of age [F(1,32) = 4.65, *p* = 0.039, η^2^ = 0.13]. The complementary analysis performed with data split by age indicated that the neonatal condition exclusively affected PINK expression at pnd 20 [F(1,16) = 6.63, *p* = 0.020, η^2^ = 0.29], by increasing PINK expression levels, in the absence of any significant effects at pnd 13. In the HCF, the analysis of the expression levels of Parkin (Figure 8C,D) showed significant interaction between factors [sex by neonatal condition by age: F(1,32) = 22.07, *p* < 0.001, η^2^ = 0.41; sex by neonatal condition: F(1,32) = 12.70, *p* = 0.001, η^2^ = 0.28; sex by age: F(1,32) = 68.62, *p* < 0.001, η^2^ = 0.68], as well as significant general effects for the sex [F(1,32) = 7.20, *p* = 0.01, η^2^ = 0.18] and age [F(1,32) = 12.14, *p* = 0.001, η^2^ = 0.28] factors. When analysing Parkin expression levels by age, we found a significant sex by neonatal condition interaction [F(1,16) = 48.27, *p* < 0.001, η^2^ = 0.75], as well as a significant general effect of the sex factor [F(1,16) = 85.06, *p* < 0.001, η^2^ = 0.84] at pnd 13, while only a significant general effect of sex was observed at pnd 20 [F(1,16) = 12.13, *p* = 0.003, η^2^ = 0.43]. Taken together, in the HCF, MD induced, in the short term (pnd 13), a significant but opposite effect on Parkin expression: an increase among males, but a decrease in females. Sex differences emerged at pnd 20 (no differences were observed at pnd 13), with female animals showing higher expression levels of Parkin than their male counterparts.

## 4. Discussion

The present results further confirm the previously reported MD-induced decrease in body weight gain (see [5] for a more complete discussion). This reduction in BW gain may reflect the transitory lack of milk ingestion (during the 24 h deprivation episode), although a more severe metabolic effect has been reported in these MD animals [44]. Changes in maternal behaviour may also affect the health status of the offspring. However, we found a compensatory increase in nursing behaviours towards the MD pups after reunion, on pnd 10, which somehow persisted the day after (pnd 11). The present results are in accordance with previous results from our research group in which MD resulted in enhanced maternal behaviour (licking–grooming frequency) after reuniting the pups with the dam [38]. More recently, an increase in active maternal behaviour upon pup reunion has been reported in rats following almost any maternal separation protocol [45]. Most probably, this increase in maternal behaviour may attempt to overcompensate for the time spent off-nest during the MD episode, and it has been reported to ‘buffer’ some adverse consequences of early stress [46]. In the present study, MD increased the latency to perform the RR at pnd 10, significantly only in males, despite a similar trend being observed among females. These observations seem to be in accordance with the results initially presented by Ellenbroek and colleagues [47], in which MD induced a delay in normal (motor) development that might also be affected by the general health status of these MD animals, which were not only deprived of maternal attention but also of food and thermic regulation at the time of testing. Indeed, postnatal underfeeding seems to be critical for reflex ontogeny [48], and MD was reported to induce short-term hypoglycaemia [44]. It is also worth mentioning that the time frame selected for the evaluation of this neuromotor reflex might be far from ideal [49], and controversial results have been reported in this regard [40,41], probably due to differences in the severity of the maternal separation procedure investigated. Further investigation is needed to better characterize the development and maturation of postnatal motor reflexes in response to an episode of ELS during the first week of postnatal life.

Herein, we have demonstrated that MD induced changes in brain cytokines in a time-and-brain-region-specific manner. A transient increase in pro-inflammatory cytokines (IL-1β and IL-6) was observed in the PFC, probably due to an immediate response to local stressful stimuli. Actually, brain IL-1β levels seem to be regulated by glucocorticoids and norepinephrine release in a sex-dependent manner [50]. In contrast, in the HCF, a long-lasting increase in IL-10 levels was reported, probably due to a compensatory effect elicited to protect this brain region from stress-induced neurodegenerative effects. Fluctuations in inflammatory markers, such as IL-1β, IL-6, and TNFα, have been previously described in neonate pups following ELS, although the results achieved are controversial [21,22,23]. Notwithstanding, such discrepancies may result from procedural differences between studies: Firstly, by differences in the nature, duration, and frequency of the stressful event to which animals were exposed [ELS due to: MD (prolonged single episode), repeated maternal separation (RMS: 3 h/day), or prenatal restraint]. In a recent systematic review, an increase in TNFα, IL-6, and IL-10 in non-blood tissues was the most commonly achieved short-term effect of ELS, i.e., RMS, considering measurements assessed less than 3 weeks after the conclusion of the stress protocol [51]. Secondly, by the time at which neuroinflammatory parameters were measured. Indeed, we have reported a decrease in hippocampal IL-1β levels in male rats immediately after MD (pnd 10) [24], but we suggested a developmental reorganization of the IL-1β system critically influencing excitatory glutamatergic synapses [24]. Notably, the role played by these stress-induced pro-inflammatory cytokines in re-sculpting brain circuitries during development seems to be brain-region-specific and sex-dependent. It is worth mentioning glial cells as the potential source of such cytokines and chemokines, although we are not able to discriminate between astrocytes and microglial cells in this regard. Reactive gliosis, which is an increase in brain GFAP expression, has been consistently reported in the short term (pnd 13) following MD, mainly in male animals [10,13], as well as in the long term [9,11,52]. Indeed, in a recent review, mother–infant disruption, both MD and maternal separation, has been demonstrated to induce an increase in GFAP expression a few days or weeks after the end of the stressful protocol [53]. Similarly, activation of microglial cells has been described following ELS, i.e., repeated maternal separation [22,23]. However, research is still needed to evaluate possible changes in microglial cells shortly after the MD episode, and thus to disentangle the origin of pro-inflammatory and anti-inflammatory cytokine fluctuations in response to MD. It would be more interesting to further investigate whether a long-lasting sensitization of the brain to the stress-inducing mechanisms occurs, as already suggested [49], following MD.

Regarding mitochondrial density, MD induced a decrease in CS activity in the PFC at the two time points analysed. In neonatal animals, a transient reduction in CS activity has been reported after mild perinatal asphyxia [54] and ouabain administration [55]. Thus, the changes in CS activity reported here might be transient and reflect a short-term adaptive response to the developmental insult, i.e., MD. The already described pro-inflammatory environment due to MD might be related to these changes in mitochondrial density, since decreases in brain CS activity have been reported in animal models of sepsis [56]. Interestingly, it is important to note that a decrease in mitochondrial density in the PFC after MD was observed in the absence of changes in mitochondrial respiratory activity, since no significant changes in CIV activity were observed among experimental groups. As an explanation, it would be plausible to consider MD to be associated with an increased number of damaged mitochondria and, therefore, with increased mitophagy activity. Mitochondrial damage in the CNS has been related to sepsis-induced inflammation; similarly, ELS-induced inflammation might alter brain mitophagy [56]. Our results revealed significant changes in the expression of PINK and Parkin proteins involved in mitophagy [57]. The analysis of mitophagy protein expression showed that PINK expression levels were only affected by MD in the long term in the HCF, while Parkin expression levels were influenced by MD in a sex- and region-dependent manner in the short term: MD induced a decrease in Parkin expression levels among female animals in the two brain regions analysed, whereas, among males, MD increased Parkin expression levels exclusively in the HFC. Increasing evidence demonstrates sex differences in mitochondrial function, particularly in the context of CNS injury. Mechanisms underlying mitochondrial biogenesis in response to brain damage seem to be sexually dimorphic, and although few studies have investigated sex differences in mitochondrial dynamics, available data suggest a differential response between males and females [58]. Moreover, sexually dimorphic temporal dynamics of maturational trajectories have been described for specific brain regions within corticolimbic circuits in rodents. Such developmental sex differences might be transient and do not necessarily persist beyond adulthood. Indeed, a greater hippocampal cell proliferation seems to take place early in life among males compared to females, probably coordinated with microglial phagocytic capacity [59]. These differences in metabolic requirements between sexes, at specific critical windows, may account, at least partially, to the reported differences. Prenatal stress has been shown to induce changes in the morphology of neuronal mitochondria [60]. Indeed, glucocorticoids critically affect the expression of mitochondrial genes [61], including Parkin expression in the frontal cortex [62]. Therefore, these mitochondrial modifications might be mediated by the MD-induced increase in plasma CORT levels and/or changes in brain GR expression [8,12]. Similarly, BDNF is known to promote mitochondrial function [63], and MD was reported to affect BDNF expression in adulthood [64], offering an alternative underlying mechanism, since decreasing BDNF expression can potentially affect mitochondrial abundance. Therefore, changes in BDNF-GR signalling can promote mitochondrial dysfunction [65], since mitochondria seem to orchestrate stress-signalling pathways by adjusting their activity to suit the prevailing energetic demands [66]. However, it is worth noting that the changes observed in PINK and Parkin expression as a response to MD are not completely consistent, possibly due to the fact that PINK does not necessarily require Parkin to activate mitophagy [67,68] and given a putative Parkin-independent mitophagy pathway [69]. In addition, changes in PINK and Parkin may not only imply defective mitophagy, since they have also been involved in neuroinflammation [27,70]. Interestingly, there is evidence implicating the PINK and Parkin mitophagy axis in inflammatory signalling, which is particularly relevant to neuronal homeostasis and function [71].

## 5. Conclusions

Early life stress, in the form of MD, altered brain inflammation processes differentially in the PFC and the HCF. A transient pro-inflammatory state was described within the PFC, whereas in the hippocampal formation, an increase in the content of IL-10, an anti-inflammatory cytokine, was described. Such alterations might be related to brain metabolism, and thus mitochondrial dynamics. Herein, we described a general decrease in mitochondria density without changes in respiratory function; thus, mitophagy was investigated, although no clear results were achieved in this regard. PINK and Parkin expression might be partially involved, although changes in PINK expression appear to be hippocampus-specific, whereas MD effects on Parkin expression seem to be sex-dependent and may not only mediate mitochondrial dynamic pathways but also a relationship with the innate immune system. For a more complete and comprehensive understanding of the effects of ELS, i.e., MD, on the developing brain, a precise identification of effects on the different cell populations is needed (neurons, astrocytes, and microglia), together with the description of a temporal profile of immediate and short- and long-term mechanisms that may sequentially appear to enable adaptation to the environment, probably opening windows for recovery and/or long-lasting damage.

## Figures and Tables

**Table 1:** Antibodies used in the present study.

Primary Antibodies			Secondary Antibodies		
Antibody	Company	Dilution	Antibody	Company	Dilution
Rabbit anti-Parkin monoclonal (48 kDa)	Thermo Fisher (702785)	1:1000	Anti-Rabbit IgG	Sigma-Aldrich (A0545)	1:2000
Rabbit anti-PINK1 polyclonal (63 kDa)	Thermo Fisher (PA1-16604)	1:1000			
Mouse anti-β-Actin monoclonal (42 kDa)	Sigma-Aldrich (A5441)	1:1500	Anti-Mouse IgG	Santa Cruz (SC-2031)	1:2500

The molecular weight of each protein is shown in parentheses (kDa). The company name includes the reference number of each antibody. IgG, Immunoglobulin G.

**Table 2:** Results of animals’ performance in the righting reflex (RR) test.

	Males		Females	
Age	CTRL	MD	CTRL	MD
**pnd 9**	1.7 ± 1.1	1.9 ± 1.0	1.7 ± 0.6	1.7 ± 0.6
**pnd 10**	1.4 ± 0.8	1.6 ± 0.5 *	1.6 ± 0.9	1.8 ± 0.8
**pnd 12**	1.1 ± 0.3	1.1 ± 0.4	1.1 ± 0.4	1.0 ± 0.2

Data represent the mean value ± the standard deviation (SD) of the latency (in seconds) that animals employed to acquire the normal prone position. Rats were exposed to early life stress, MD (24 h on pnd 9) or subjected to the same manipulation except for the separation period (CTRL). Pups were individually tested before the procedure (pnd 9), immediately after (pnd 10), and on pnd 12 (n = 12 per experimental group). Two-way ANOVA (pnd 9 and 12) or Kruskal–Wallis followed by Mann–Whitney comparisons (pnd 10): * *p* < 0.05 vs. CTRL male.

**Table 3:** Observation of maternal behaviours regarding their corresponding litters.

	Maternal Contact		Maternal Nursing		Non-Maternal Care Behaviour	
Age	CTRL	MD	CTRL	MD	CTRL	MD
**pnd 10**	6.50 ± 2.26	8.83 ± 1.60	5.50 ± 1.87	9.00 ± 1.79 ^b^	4.17 ± 2.48	2.17 ± 1.72
**pnd 11**	2.67 ± 4.08 ^c^	4.17 ± 1.83 ^c^	1.17 ± 1.60 ^c^	4.00 ± 2.36 ^bc^	5.83 ± 3.19 ^c^	5.17 ± 1.72 ^c^

Data represent the mean value ± the standard deviation (SD) of the frequency of maternal contacts established, the time spent devoted to maternal nursing behaviours, and the time spent on other activities. Rats were exposed to early life stress, MD (24 h on pnd 9), or submitted to the same manipulation except for the separation period (CTRL). The behaviour of each dam was investigated immediately after reunion (pnd 10) and the day after (pnd 11) (n = 3 litters per experimental group). Two-way ANOVA: ^b^, *p* < 0.05, significant overall effect of neonatal condition; ^c^, *p* < 0.05, significant overall effect of day.

**Table 4:** Principle maternal deprivation (MD) effects on pro-inflammatory and anti-inflammatory cytokines and chemokines obtained from multiplex analysis.

	Analytes	PFC		HCF	
Age		pnd 13	pnd 20	pnd 13	pnd 20
**Cytokines:** **Pro-inflammatory**	**IL-1β**	↑	=	=	=
	**IL-6**	↑	=	=	=
	**TNFα**	=	=	=	=
**Anti-inflammatory**	**IL-10**	=	=	↑	↑
**Chemokines**	**CX3CL1**	=	=	=	=
	**CCL2/MCP1** **MCP-1/CCL2**	n.d.	n.d. males; ↓ (only females)	n.d.	n.d.

Analytes were measured by a multiplex analysis in the prefrontal cortex (PFC) and hippocampal formation (HCF) of rats exposed to early life stress, MD (24 h on pnd 9), or subjected to the same manipulation except for the separation period. The reported changes due to MD are indicated: ↑, increase in levels; ↓, decrease in levels; =, no changes; n.d., levels not determined. Abbreviations: Interleukin 1beta (IL-1β), Interleukin 6 (IL-6), Tumor Necrosis Factor Alpha (TNFα), Interleukin 10 (IL-10), Fractalkine/CX3CL1, Monocyte Chemoattractant Protein-1 (MCP-1/CCL2). Only statistically significant results are indicated; sex and age differences are not shown in the table (see text for details).

**Table 5:** Main maternal deprivation (MD) effects on the mitochondrial dynamic parameters evaluated.

		PFC		HCF	
		pnd 13	pnd 20	pnd 13	pnd 20
**Enzymatic activity:**					
	**Citrate Synthase (CS)**	↓	↓	=	=
	**Complex IV (CIV)**	=	=	=	=
	**Ratio CIV/CS**	↑	↑	=	↑
**Protein Expression (WB):**					
	**PINK**	=	=	=	↑
	**Parkin**	↓ (only in females)	=	↑ in males ↓ in females	=

Enzymatic activity was measured by colorimetric assays, whereas protein expression was analysed using Western blotting (WB). Analyses were performed in the prefrontal cortex (PFC) and hippocampal formation (HCF) of rats exposed to early life stress, MD (24 h on pnd 9), or submitted to the same manipulation except for the separation period. The reported changes due to MD are indicated: ↑, increase; ↓, decrease; or =, no change. Only statistically significant results are indicated; sex and age differences are not shown in the table (see text for details).

## Data Availability

The data presented in this study are available upon request from the corresponding author.

## Supplementary Materials

The following supporting information can be downloaded at: https://www.mdpi.com/article/10.3390/biology13121011/s1, Example of the original Western blot images.
