# Supplementary material for: Consequences of Early Maternal Deprivation on Neuroinflammation and Mitochondrial Dynamics in the Central Nervous System of Male and Female Rats
Source: Biology (Basel). 2024 Dec 4;13(12):1011. doi: 10.3390/biology13121011 (PMC11672930; doi:10.3390/biology13121011)
Supplement: Supplementary file 1 [file biology-13-01011-s001.zip › biology-3312409-supplementary.pdf]

PARKIN hippocampal formation

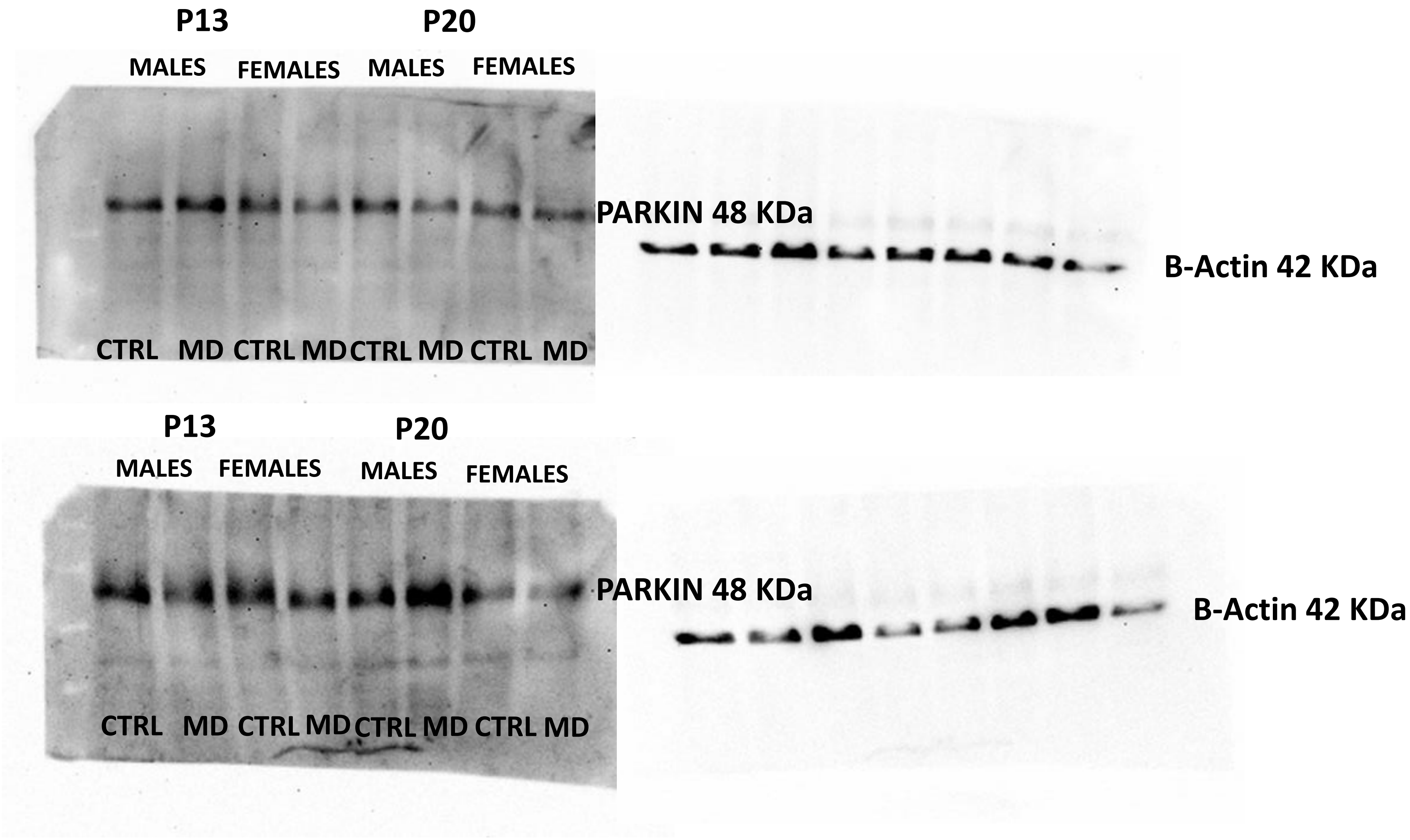
P13
P20
MALES
FEMALES
MALES
FEMALES
PARKIN 48 KDa
B-Actin 42 KDa
CTRL MD CTRL MD CTRL MD CTRL MD
P13
P20
MALES
FEMALES
MALES
FEMALES
PARKIN 48 KDa
B-Actin 42 KDa
CTRL MD CTRL MD CTRL MD CTRL MD

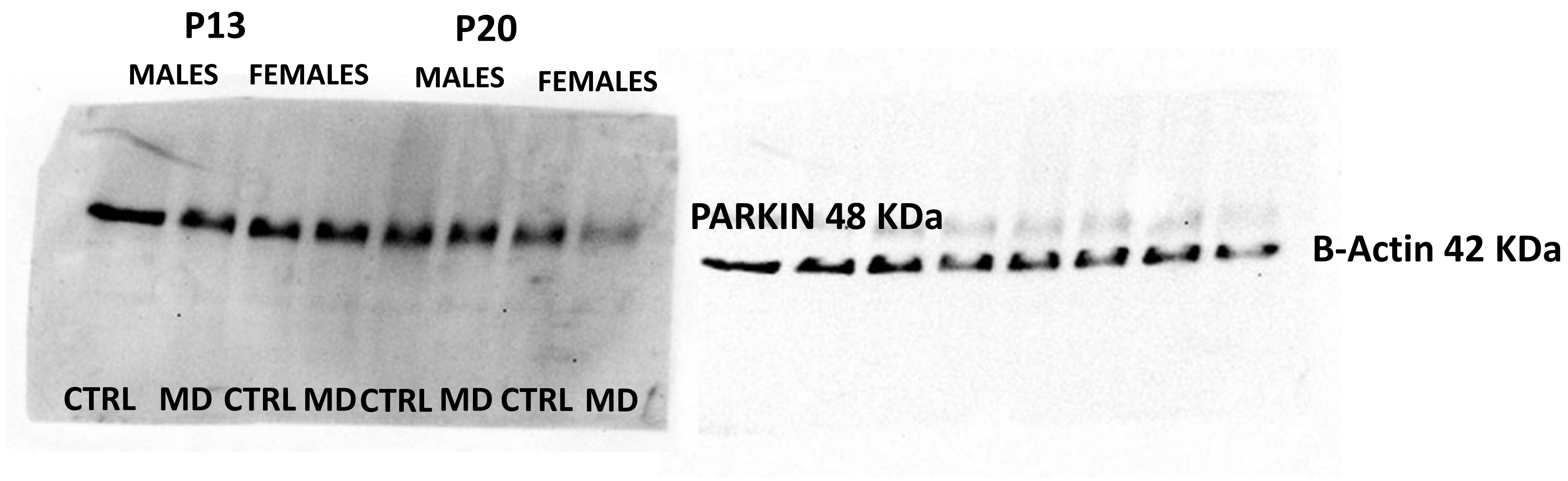
P13
P20
MALES
FEMALES
MALES
FEMALES
PARKIN 48 KDa
B-Actin 42 KDa
CTRL
MD
CTRL
MD
CTRL
MD
CTRL
MD

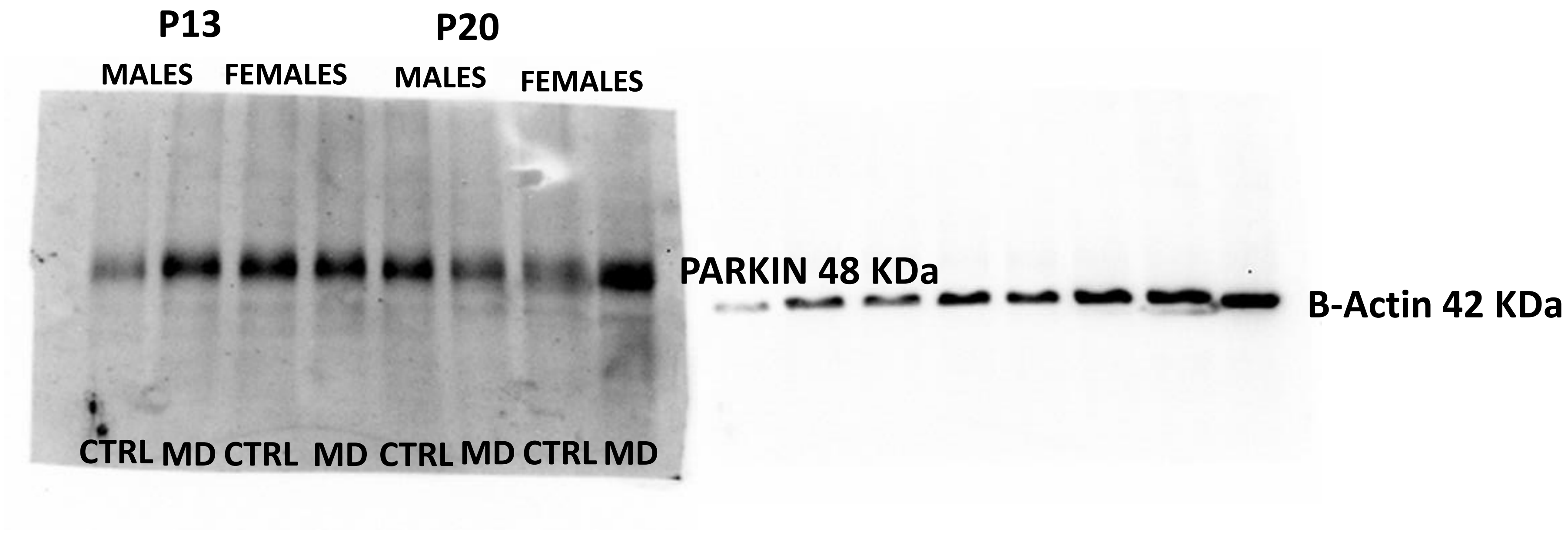
P13
P20
MALES
FEMALES
MALES
FEMALES
PARKIN 48 KDa
B-Actin 42 KDa
CTRL
MD
CTRL
MD
CTRL
MD
CTRL
MD

PARKIN prefrontal cortex

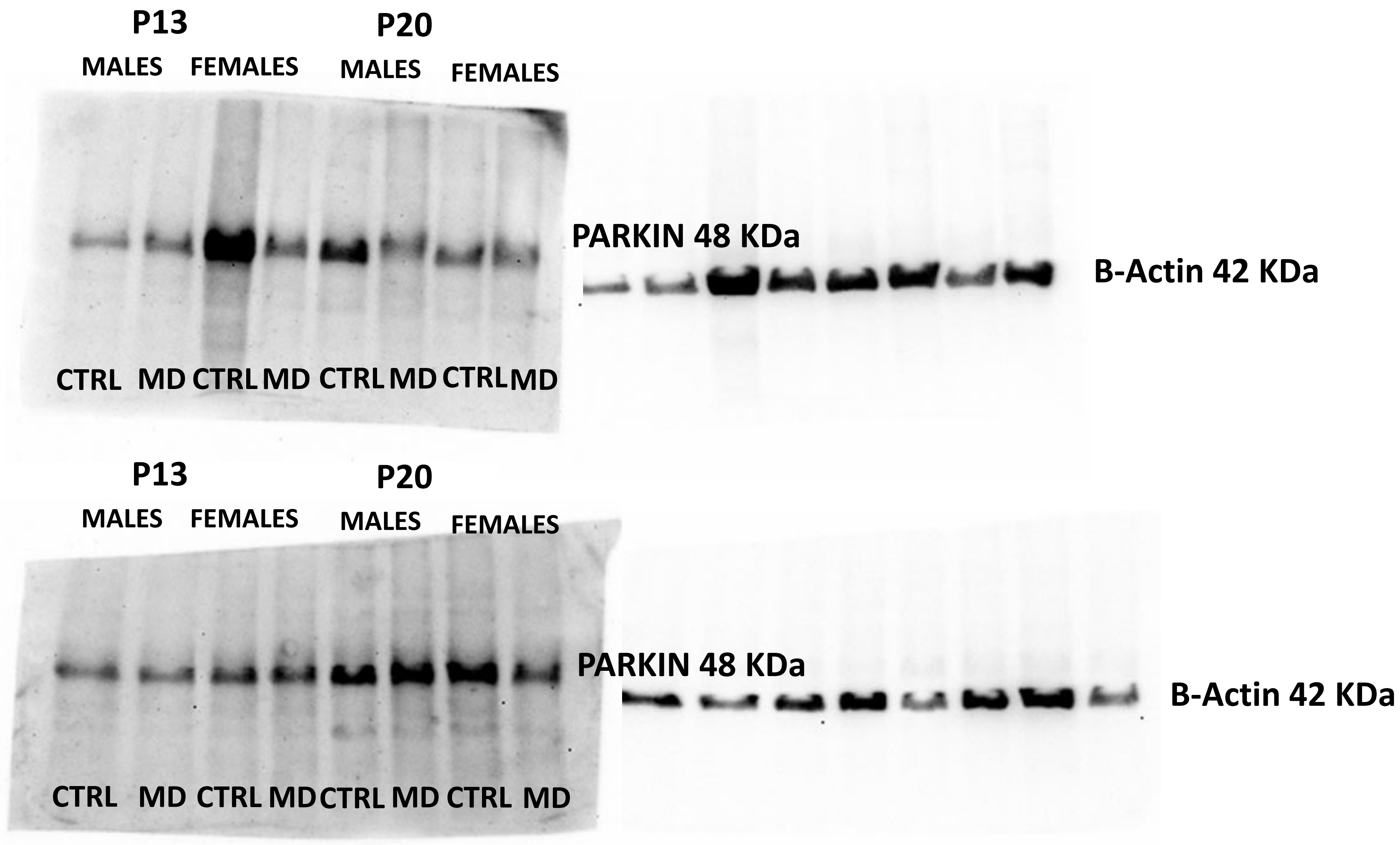
P13
P20
MALES
FEMALES
MALES
FEMALES
PARKIN 48 KDa
B-Actin 42 KDa
CTRL
MD
CTRL
MD
CTRL
MD
CTRL
MD
P13
P20
MALES
FEMALES
MALES
FEMALES
PARKIN 48 KDa
B-Actin 42 KDa
CTRL
MD
CTRL
MD
CTRL
MD
CTRL
MD

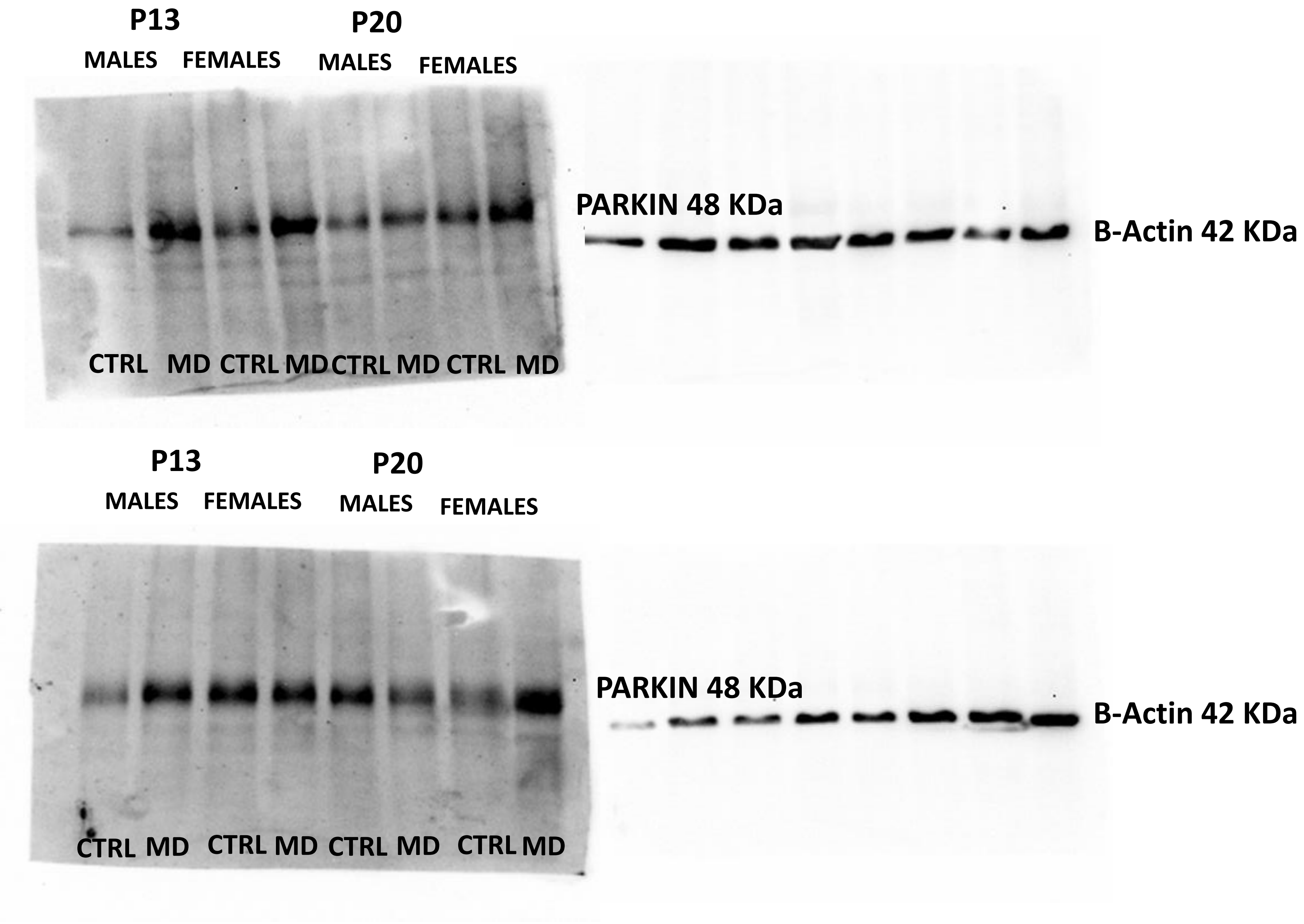
P13
MALES
FEMALES
P20
MALES
FEMALES
PARKIN 48 KDa
B-Actin 42 KDa
CTRL MD CTRL MD CTRL MD CTRL MD
P13
MALES
FEMALES
P20
MALES
FEMALES
PARKIN 48 KDa
B-Actin 42 KDa
CTRL MD CTRL MD CTRL MD CTRL MD

PINK hippocampal formation

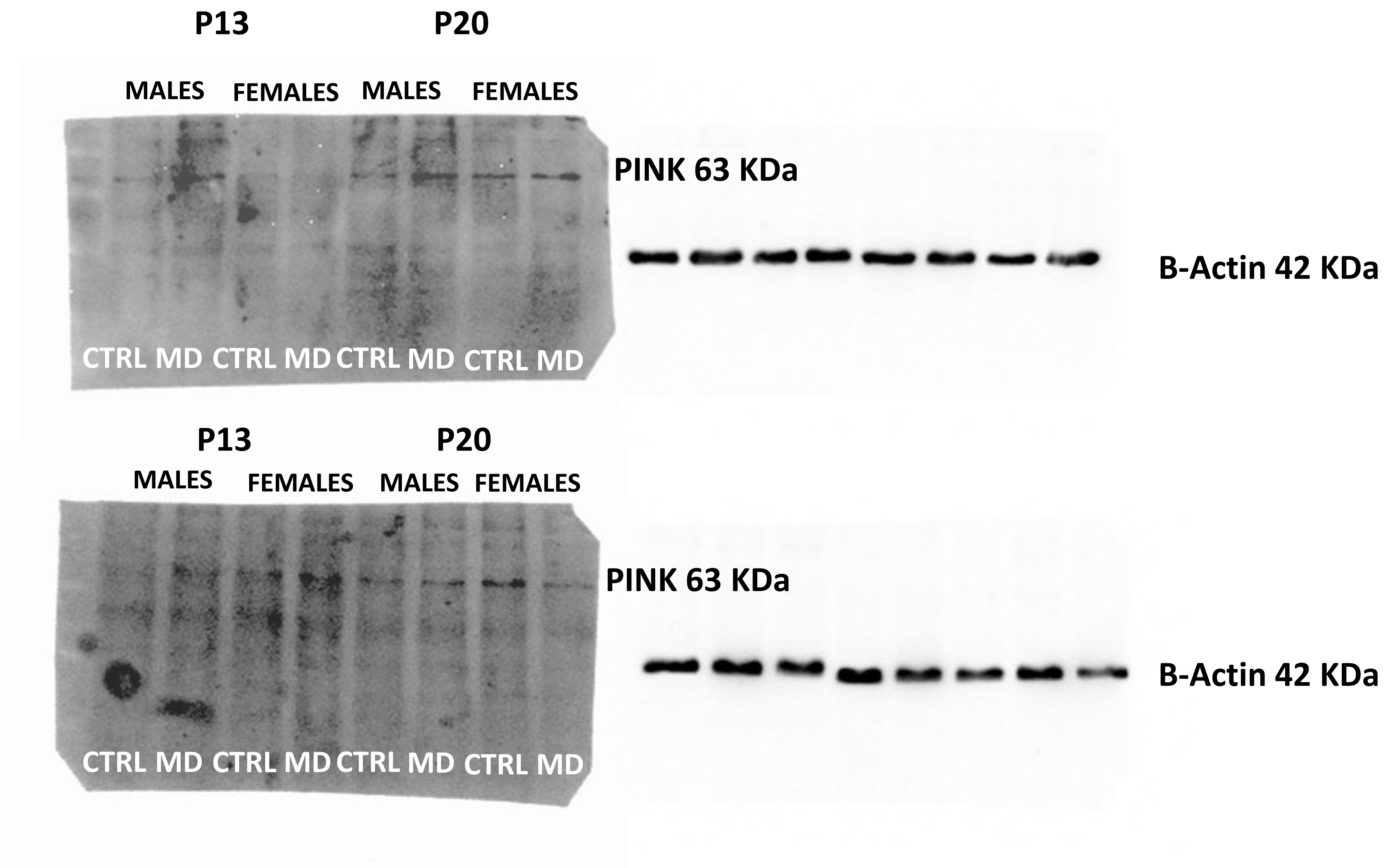

P13
P20
MALES
FEMALES
MALES
FEMALES
PINK 63 KDa
B-Actin 42 KDa
CTRL MD CTRL MD CTRL MD CTRL MD
P13
P20
MALES
FEMALES
MALES
FEMALES
PINK 63 KDa
B-Actin 42 KDa
CTRL MD CTRL MD CTRL MD CTRL MD

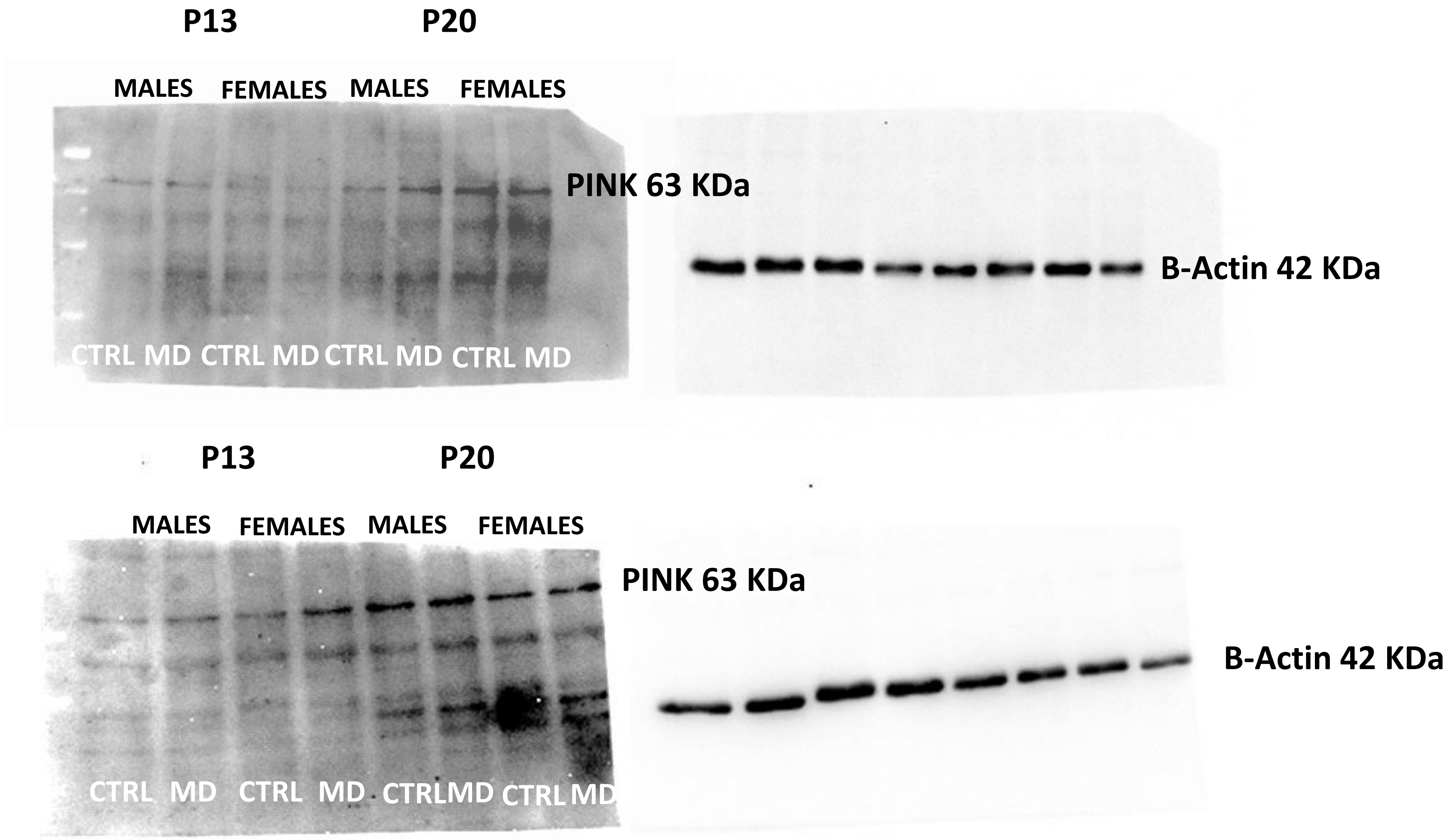
P13
P20
MALES
FEMALES
MALES
FEMALES
PINK 63 KDa
CTRL MD CTRL MD CTRL MD CTRL MD
B-Actin 42 KDa
P13
P20
MALES
FEMALES
MALES
FEMALES
PINK 63 KDa
CTRL MD CTRL MD CTRLMD CTRL MD
B-Actin 42 KDa

PINK prefrontal cortex

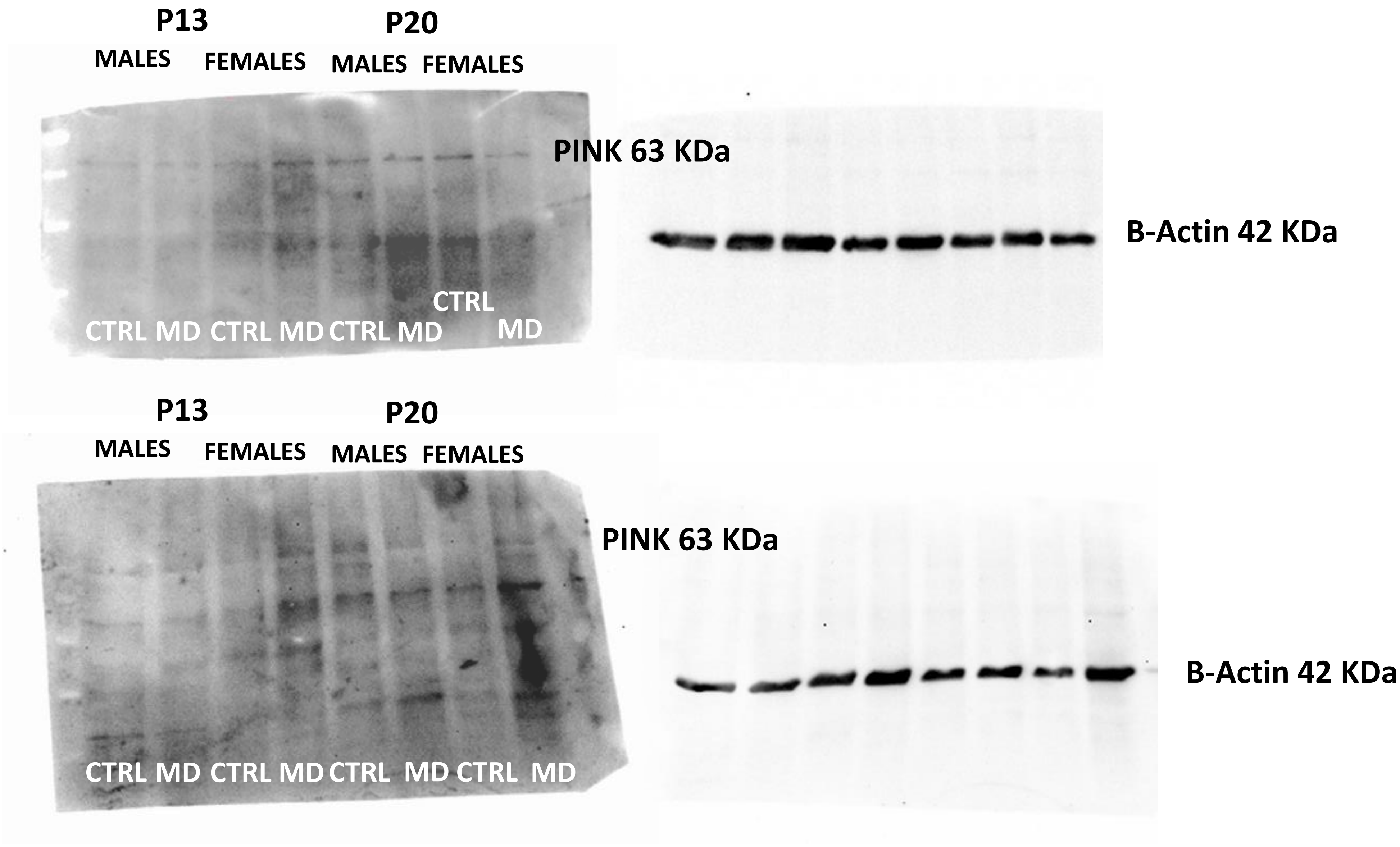
P13
P20
MALES
FEMALES
MALES
FEMALES
PINK 63 KDa
B-Actin 42 KDa
CTRL MD CTRL MD CTRL MD CTRL MD
P13
P20
MALES
FEMALES
MALES
FEMALES
PINK 63 KDa
B-Actin 42 KDa
CTRL MD CTRL MD CTRL MD CTRL MD

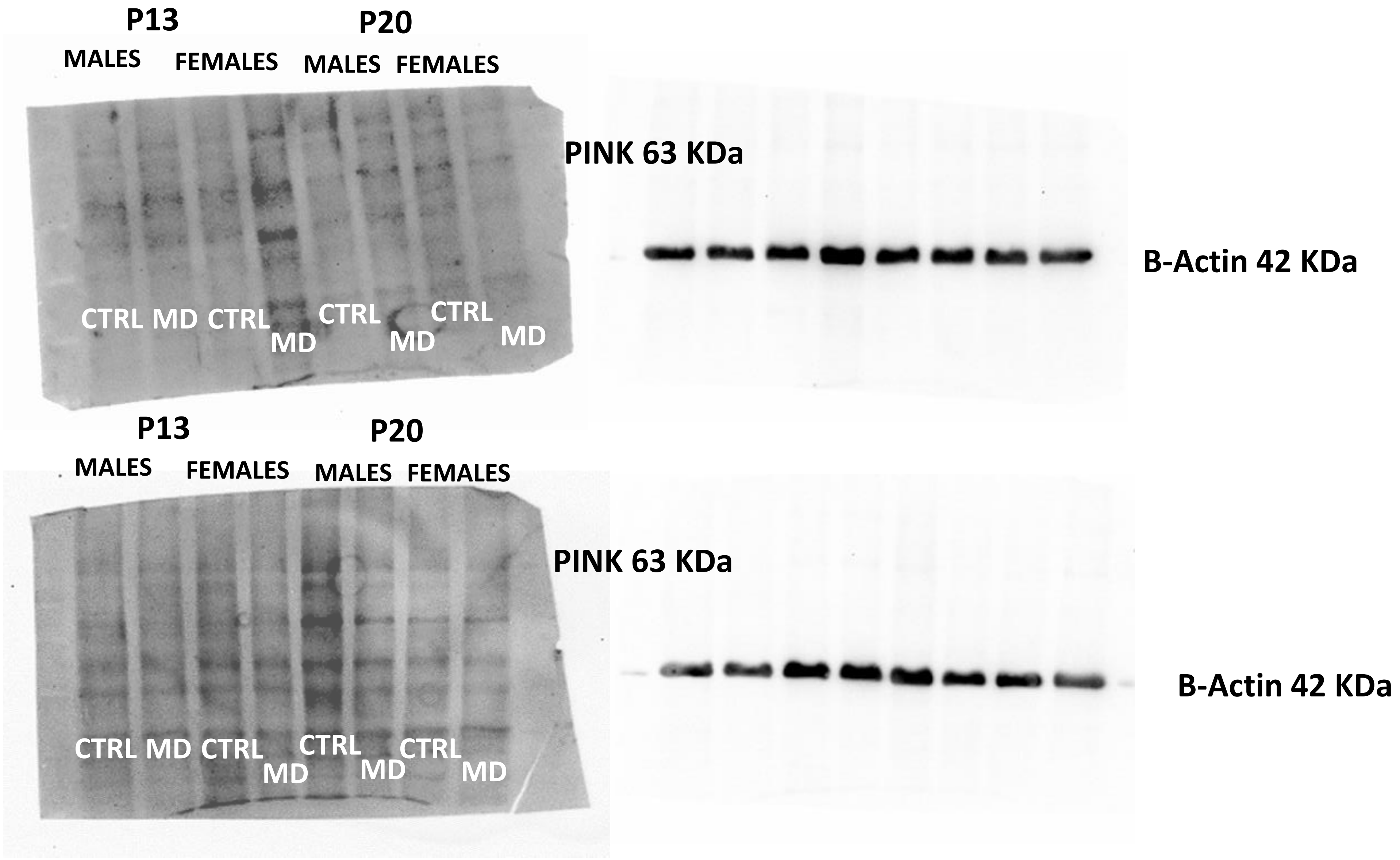
P13
P20
MALES
FEMALES
MALES
FEMALES
PINK 63 KDa
CTRL MD CTRL MD CTRL MD CTRL MD
B-Actin 42 KDa
P13
P20
MALES
FEMALES
MALES
FEMALES
PINK 63 KDa
CTRL MD CTRL MD CTRL MD CTRL MD
B-Actin 42 KDa
